# Best practice management guidelines for fibrous dysplasia/McCune-Albright syndrome: a consensus statement from the FD/MAS international consortium

**DOI:** 10.1186/s13023-019-1102-9

**Published:** 2019-06-13

**Authors:** Muhammad Kassim Javaid, Alison Boyce, Natasha Appelman-Dijkstra, Juling Ong, Patrizia Defabianis, Amaka Offiah, Paul Arunde, Nick Shaw, Valter Dal Pos, Ann Underhil, Deanna Portero, Lisa Heral, Anne-Marie Heegaard, Laura Masi, Fergal Monsell, Robert Stanton, Pieter Durk Sander Dijkstra, Maria Luisa Brandi, Roland Chapurlat, Neveen Agnes Therese Hamdy, Michael Terrence Collins

**Affiliations:** 1 Nuffield Department of Orthopaedics, Rheumatology and Musculoskeletal Sciences, University of Oxford, Oxford, UK; 2 Skeletal Disorders and Mineral Homeostasis Section, National Institute of Dental and Craniofacial Research, Bethesda, MD USA; 3 Department of Medicine, Division of Endocrinology & Center for Bone Quality, Leiden University Medical Center, Leiden, The Netherlands; 4 Department of Plastic Surgery, Craniofacial Centre, Great Ormond Street Hospital for Children NHS Trust, London, UK; 5 Section of Paediatric Dentistry, University of Turin, Turin, Italy; 6 Department of Oncology & Metabolism, University of Sheffield, Sheffield, UK; 7 Metabolic Bone Team, Sheffield Children’s Hospital, Sheffield, UK; 8 Endocrine Department, Birmingham Women’s and Children’s NHS Foundation Trust, Birmingham, UK; 9 European Association of Friends of McCune-Albright Syndrome (TO), Turino, Italy; 10 Fibrous Dysplasia Support Society, Birmingham, UK; 11 Fibrous Dysplasia Foundation, Grandville, USA; 12 Department of Drug Design and Pharmacology, University of Copenhagen, Copenhagen, Denmark; 13 Department of Surgery and Translational Medicine, University of Florence, Florence, Italy; 14 Paediatric Orthopaedic and Trauma Surgery, University Hospitals Bristol NHS Foundation Trust, Bristol, UK; 15 Department of Orthopaedic Surgery, Nemours Children’s Hospital, Orlando, Florida USA; 16 Department of Orthopaedic Surgery, Leiden University Medical Center, Leiden, The Netherlands; 17 INSERM UMR 1033 and Université de Lyon, Lyon, France

**Keywords:** Fibrous dysplasia, McCune Albright syndrome, Guidelines, Diagnosis, Management

## Abstract

Fibrous Dysplasia / McCune Albright syndrome (FD/MAS) represents a wide spectrum of diseases due to somatic gain-of-function mutations of the *GNAS* gene. The mutation leads to overactivity in the target tissues and to a wide phenotype of clinical features that vary in severity and age of onset. The rarity of the disease and its variable presentation to multiple specialities often leads to misdiagnosis and inappropriate variability in investigations and treatments. To address this, our international consortium of clinicians, researchers, and patients’ advocates has developed pragmatic clinical guidelines for best clinical practice for the definition, diagnosis, staging, treatment and monitoring for FD/MAS to empower patients and support clinical teams in both general and specialised healthcare settings. With the lack of strong evidence to inform care, the guidelines were developed based on review of published literature, long-standing extensive experience of authors, input from other healthcare professionals involved in the care of FD/MAS patients and feedback from patients and patient groups across the globe. This has led to the formulation of a set of statements to inform healthcare professionals, patients, their families, carers and patient groups of the best practice of care. It is anticipated the implementation of these recommendations will lead to improvement in the care of patients with FD/MAS internationally.

**Electronic supplementary material:**

The online version of this article (10.1186/s13023-019-1102-9) contains supplementary material, which is available to authorized users.

## Background

Fibrous dysplasia/McCune-Albright syndrome (FD/MAS; OMIM#174800) is a rare disorder characterized by skeletal lesions, skin hyperpigmentation, and hyper-functioning endocrinopathies [1, 2]. It arises from post-zygotic gain-of-function mutations in the *GNAS* gene, which encodes the α-subunit of the G_s_ signalling protein [3]. These mutations disrupt the intrinsic GTPase activity of G_s_α, leading to persistent stimulation of adenylyl cyclase and dysregulated production of cyclic AMP and downstream signalling [4]. The resulting disease is mosaic with a broad clinical spectrum, ranging from a trivial incidentally discovered radiographic finding to severe and disabling disease. FD may involve one (monostotic) or multiple (polyostotic) bones and may occur in isolation or in combination with extraskeletal disease [5]. While FD/MAS is classically defined as involving the skeleton, skin, and endocrine systems, given the ubiquitous nature of G_s_ signalling, multiple other tissues may also be affected. Any part or combination of features may be present.

Clinical management in FD/MAS is challenging, and multiple barriers exist to providing consistent, high quality care. Some of these include the broad clinical spectrum which results in considerable phenotypic variability among patients; multisystem involvement which requires coordination between diverse specialties; and disease rarity, which makes it challenging for individual clinicians and centres to gain specialist expertise in the disorder’s ubiquitous manifestations. There is little high-quality evidence to inform the diagnosis and management of FD/MAS. There are as yet substantial knowledge gaps about FD/MAS pathophysiology and natural history, and a paucity of hard evidence from clinical trials for different diagnostics and therapeutic approaches. To address these challenges, an international consortium of clinicians, researchers, and patients’ advocates convened to develop standard of care guidelines for diagnosis and management of FD/MAS based on best available evidence and expert opinion [6].

The aim of developing best clinical practice care guidelines for diagnosis and management of FD/MAS is to harmonize the care of FD/MAS internationally, to provide standards of care for the development and evaluation of patient-related outcome measures, to provide a uniform cross-border standard of care for inclusion of patients into clinical trials, and to enable comparison of collected clinical care and research FD/MAS data between centres and studies.

## Methods

These guidelines were co-developed by clinical experts in the management of FD/MAS from the FD/MAS consortium and patient advocacy groups [6]. The FD/MAS Consortium consisted of 51 FD/MAS clinical and patient experts from 13 countries from Europe, the United States of America and Asia. The Guideline Development Group (GDG) consisted of a subgroup of experts from paediatric and adult rheumatology, endocrinology, orthopaedics, maxillofacial surgery, radiology, dentistry, a pain specialist and expert representatives form national FD/MAS patient groups. The GDG was formed at a consensus meeting held in Oxford in October 2015 [6]. This first meeting identified key questions for the development of clinical care guidelines and a modified Delphi approach was selected to address these questions. The GDG reviewed the past 30 years’ published evidence using the MeSH term “Fibrous Dysplasia of Bone” on Medline. Given the rarity of FD/MAS there are very few published randomised control trials, and marked heterogeneity in case definitions, interventions and outcomes used. The GDG therefore used the best of the existing data together with personal expertise and experience in FD/MAS.

The GDG developed a first draft of the clinical care pathway that was circulated to the broader membership of the Consortium for comment. The responses were reviewed at the second consensus meeting of the FD/MAS Consortium in Lyon in December 2016. The consensus draft of the clinical care pathways was recirculated to members of the international consortium for final comments. The comments and suggestions for the clinical pathway were then reviewed by the writing group and formulated statements with over 70% consensus were included in the final report. The patient group representatives then independently developed a separate patient checklist to give context to the clinical pathway, including questions patients may want to ask their doctor and questions their doctor may ask them.

The writing group chose to submit the guidelines for publication in an open access journal in order to make it freely accessible to a wide readership of clinicians, basic scientists and patients, also using the creative commons licence on patient group websites. The Consortium will review the guidelines at least every 5 years or sooner if required by breakthrough findings from published literature. The guidelines were developed without external financial support from industries involved in therapies for FD/MAS. Competing interests of members were recorded and documented in the pathway.

### Definition

**Fig. 1:**
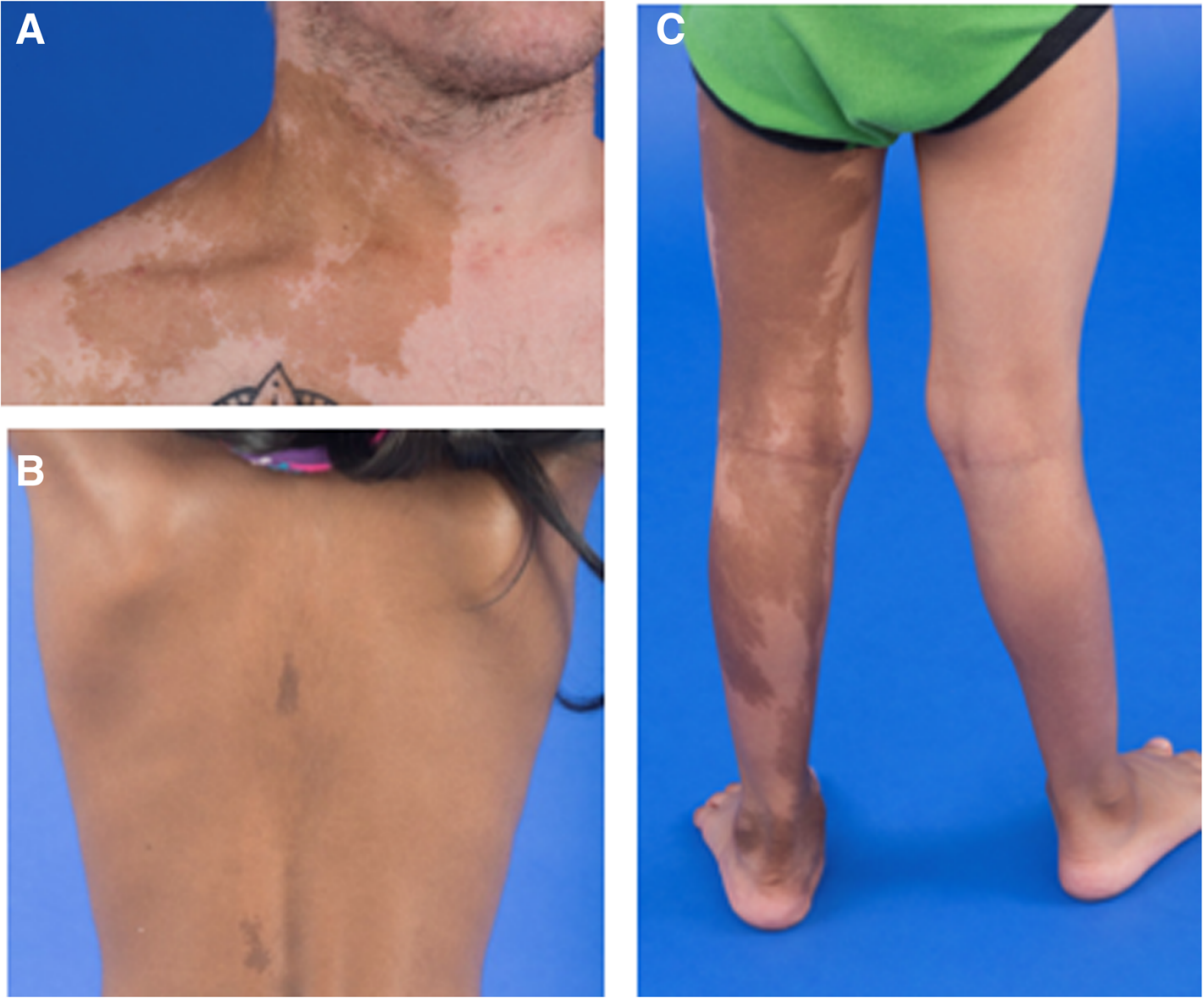
Representative images of café-au-lait macules in patients with McCune-Albright syndrome. Photographs of the shoulder (**a**), back (**b**), and legs (**c**) from three patients demonstrating characteristic hyperpigmented lesions with jagged borders, and tendency to either occur or reflect around (“respect”) the midline of the body. Images A and C show large lesions, while the patient in image B has two small lesions in a classic location, demonstrating the broad potential spectrum of involvement

**Fig. 2:**
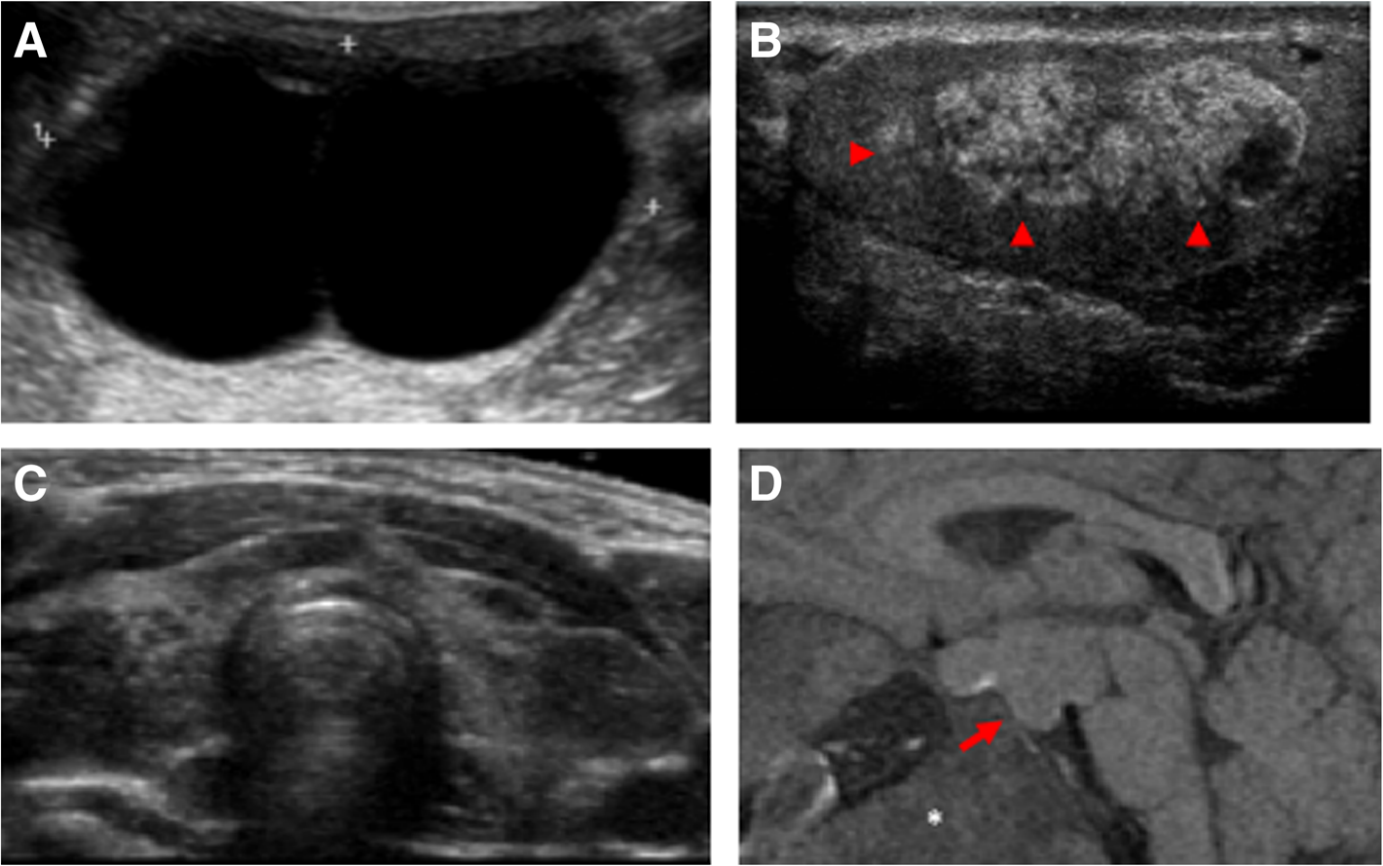
Representative radiographic features of endocrine involvement in McCune-Albright syndrome. **a** Pelvic ultrasonography in a 5-year-old girl with clinical signs of precocious puberty demonstrating a large unilateral ovarian cyst. **b** Testicular ultrasonography in a patient with macro-orchidism demonstrating a discrete, mixed hyper- and hypoechoic lesion (red arrowheads). **c** Thyroid ultrasonography showing diffuse, bilateral involvement with multiple hyper- and hypoechoic nodules. **d** A pituitary MRI in a patient with growth hormone excess revealing a pituitary macroadenoma (red arrow) and fibrous dysplasia involvement throughout the skull base (white star)

A diagnosis of the subtypes of FD/MAS can only be made after a thorough evaluation of a) the extent of skeletal disease: monostotic/polyostotic and b) the presence of extra-skeletal manifestations. Monostotic fibrous dysplasia is defined as the presence of fibrous dysplasia in one skeletal site only. Polyostotic fibrous dysplasia is defined as the presence of fibrous dysplasia in more than one skeletal site without extra-skeletal manifestations. McCune-Albright syndrome is defined as the combination of FD and one or more extra skeletal feature, OR the presence of two or more extra skeletal features. Not requiring FD for the diagnosis of MAS reflects better understanding of the molecular pathogenesis of the disorder. Mazabraud Syndrome is the combination of FD with intramuscular myxoma(s). The myxoma is defined as an extra-skeletal manifestation of FD/MAS and may occur in association with any type of the disease (monostotic, polyostotic or MAS). Other extra-skeletal features include:

1. Café-au-lait skin macules with characteristic features of jagged, irregular borders (Coast of Maine) and a distribution showing the so-called “respect of” the midline of the body (Fig. 1);
2. Gonadotropin-independent sex steroid production resulting in precocious puberty, recurrent ovarian cysts in girls (Fig. 2a) and women or autonomous testosterone production in boys and men (Fig. 2b). This includes testicular lesions consistent with FD/MAS with or without associated gonadotropin-independent precocious puberty.
3. Thyroid lesions consistent with FD/MAS with or without non-autoimmune hyperthyroidism (Fig. 2c)
4. Growth hormone excess (Fig. 2d)
5. Neonatal hypercortisolism

Of note, FGF-23-associated hypophophataemia is not considered a feature of MAS but rather a marker of the severity of skeletal FD.

### Diagnosis

In most cases, the diagnosis of FD/MAS can be made clinically after a complete staging evaluation for skeletal, endocrine, soft tissue and dermatologic features [7, 8]. Isolated monostotic bone lesions without accompanying skin or endocrine findings include a wide differential diagnosis and diagnostic uncertainty and usually require histological confirmation (Table 1). In some cases a molecular diagnosis of affected tissues is indicated when clinical, radiological and histological analysis fails to confirm the diagnosis of FD.

**Table 1:** Potential mimics of fibrous dysplasia by skeletal site

Site	Differential diagnosis
General	Cancer (primary or secondary) or hematologic malignancy including solitary plasmocytoma sarcoma
	Enchondromatosis
	Simple bone cyst (unicameral)
	Giant cell tumours
	Aneurysmal bone cyst
	Paget’s disease of bone
	Neurofibromatosis type I
	Cutaneous Skeletal Hypophosphatemia syndrome
	Langerhans cell Histiocytosis
	Melorrheostosis
	Osteonecrosis
	Osteitis Fibrosa Cystica (Recklinghausen)
Craniofacial bones	Ossifying fibroma
	Fibro-osseous lesion
	Cherubism
	Aseptic mandibular osteitis (SAPHO syndrome)
	Central giant-cell granuloma
Fronto-sphenoidal	Meningioma
Tibia	Adamantinoma and osteofibrous dysplasia

### Radiological characterisation

A number of radiological techniques are recommended for the diagnosis of FD/MAS and specialist radiological expertise is required to differentiate FD/MAS from other mimics.

**Fig. 3:**
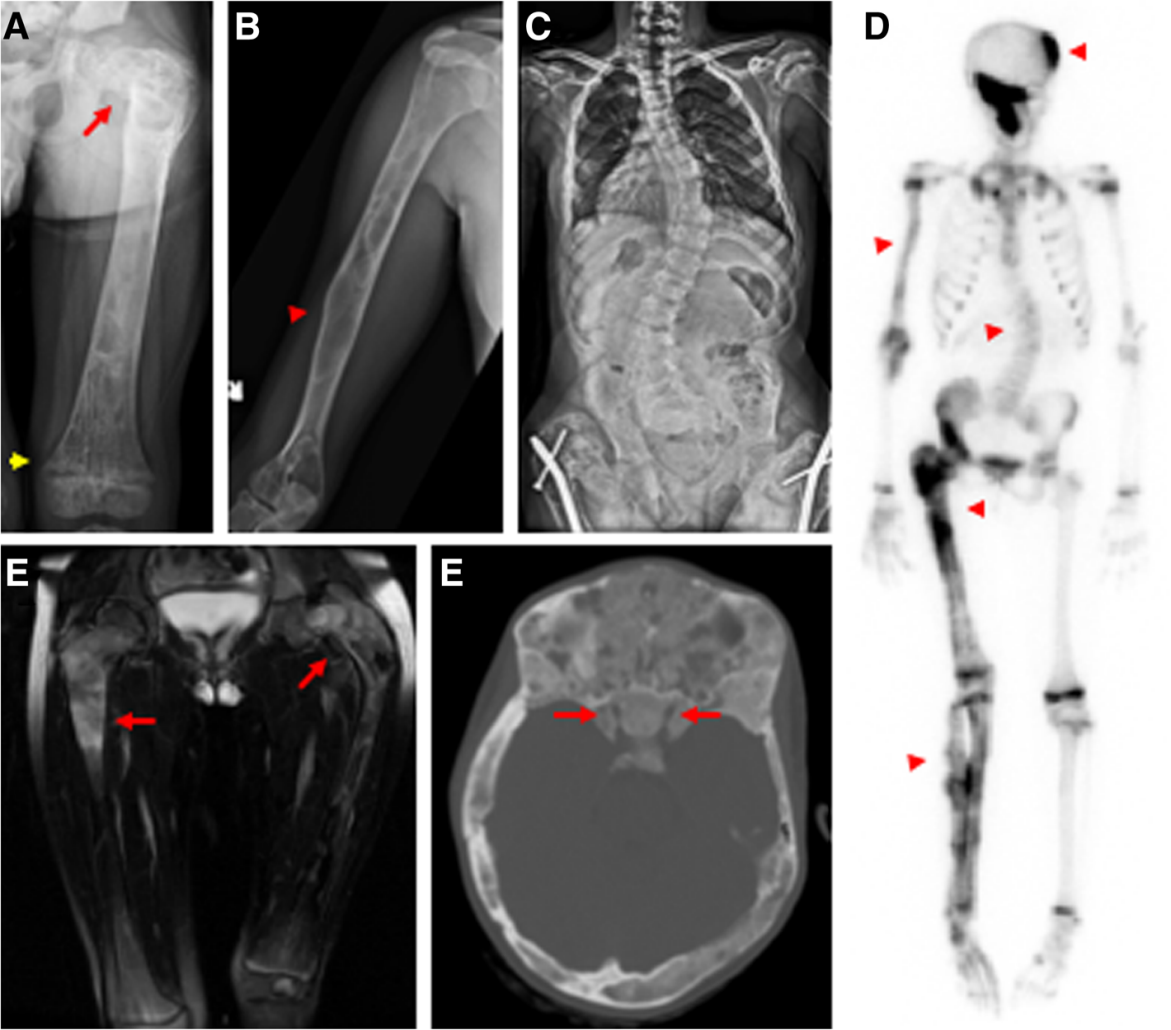
Representative radiographic features of fibrous dysplasia. **a** Femoral X-ray demonstrating diffuse involvement with fibrous dysplasia and a coxa vara (“shepherd’s crook”) deformity (red arrow). Note the irregular appearance of the distal femoral metaphyses (yellow arrowhead) resulting from FGF-23-mediated rickets. **b** Humeral X-ray demonstrating characteristic features of fibrous dysplasia, including homogenous “ground glass” appearance and cortical thinning. Bowing has occurred at a previously fractured site in the midshaft (red arrowhead). **c** X-ray from a patient with diffuse spinal FD and resulting thoraco-lumbar scoliosis. Note the presence of bilateral intramedullary femoral rods. **d** Technetium-99 scintigraphy scan showing increased tracer uptake in areas of fibrous dysplasia, including the skull, spine, right humerus, and right lower extremity (red arrowheads). Diffuse bilateral tracer uptake is also observed in the epiphyses of this growing adolescent. **e** T2-weight magnetic resonance imaging of the lower extremities showing well-demarcated lesions of intermediate to high signal intensity in the bilateral femurs (red arrows), corresponding to fibrous dysplasia lesions. **f** Computed tomography of the skull showing diffuse homogenous, “ground glass” involvement characteristic of craniofacial fibrous dysplasia. The bilateral optic canals are involved with fibrous dysplasia and widely patent (red arrows)

General radiologic features of FD/MAS on conventional radiography include: ground-glass appearance; completely radiolucent (cystic) lesions, sclerotic lesions or mixed cystic and sclerotic lesions; well-circumscribed margins (geographic pattern), with or without a sclerotic border; and expanded lesions with a shell that is thick, thin, or showing small perforations and/or endosteal scalloping [9]. A soft tissue mass is not a radiological feature of FD and should be further investigated (Fig. 3a–e). The *specific* radiological features of FD are described in Table 2.

**Table 2:** Specific radiological features dependent on body site [10]

Bone	Features
Pelvis and ribs	• Fibrous dysplasia is the most common cause of a benign expansile lesion of a rib. • Expansile lytic lesion • Fusiform enlargement of the rib • Minor calcifications within the lesion may be seen
Extremities	• Bowing deformity, in particular of the large weight-bearing bones (e.g. shepherds crook deformity of the proximal • femur) • Looser zones • Co-existent precocious puberty may lead to premature fusion of growth plates resulting in short stature
Skull and craniofacial bones	• Bone expansion showing ground-glass appearance • Calvarial deformity resulting in exophthalmos

CT is useful for assessing regions with complex anatomy of skeletal structure e.g. face, pelvis, spine, and for detecting subtle un-displaced fractures. The presence of extra-osseous soft tissue mass with bony destruction would suggest malignant transformation. Although FD has non-specific features on MRI, this imaging tool allows differentiation of FD from a cyst lesion. The lesion(s) may contain fluid/fluid levels but there should not be a soft tissue mass. Lesions are patchy with low to intermediate signalling on T1 and T2-weighted images and may show high signalling on T2-weighted images in children. Inversion recovery pulse sequences give a high signal and there is patchy contrast enhancement.

Nuclear medicine imaging studies such as ^99m^Tc-MDP, usually combined with single-photon emission computed tomography (SPECT) to give better anatomical resolution, or ^18^F NaF PET/CT demonstrate increased tracer uptake at the foci of skeletal disease [10].

### Histological and genetic characterisation

Biopsy with histological evaluation of suspected bone disease is usually only necessary in unusual or questionable cases, and/or if malignancy is suspected. The risks and benefits of a biopsy should be clearly explained to patients, including that a biopsy does not typically lead to regrowth of FD.

The benefit of genetic testing in patients with a clear clinical diagnosis is uncertain. A genetic diagnosis is recommended where the diagnosis is in question. This especially applies to isolated/monostotic lesions in the skull, after exclusion of other associated skeletal and/or extraskeletal features- e.g. other bones / skin features/ endocrinopathies. Diagnostic biopsies should be processed as fresh or fresh frozen material to enable genetic testing for *GNAS* mutation. False negatives may occur if the biopsy contains normal tissue and the biopsy may need to be repeated. Mutation analysis can also be performed in paraffin-embedded samples although false negatives are then more likely [11]. Next generation sequencing (NGS) has a lower false negative outcome than Sanger sequencing. False positives have not been described using NGS and this sequencing technique can be used to differentiate FD/MAS from osteosarcomas [12]. Use of blood for mutation analysis cannot exclude the diagnosis of monostotic FD, but a positive result is informative.

## Staging of FD/MAS

The purpose of staging of FD/MAS is to determine the full extent and impact of disease at diagnosis to guide tests and treatments and to minimize risk of complications. Staging should be considered at the time of presentation with suspected FD/MAS taking into account age and clinical presentation. The key components of staging are listed below.

### Evaluation of the skeletal system

(See Additional file 2: Flow chart: Skeletal evaluation FD lesion(s))

Assessment of the skeletal system requires a full medical history, physical examination, laboratory investigations and radiological and nuclear imaging. Assessment of skeletal symptoms should include a skeletal map with areas marked for bone pain (see below), joint pain and bone/ joint deformity; a fracture history including site, date, level of trauma (e.g. spontaneous, fragility, severe trauma) and fracture healing (complete / incomplete / non-healing); previous orthopaedic procedures (type and date) including details of metalwork insertion (location and type). The following information should be collected on previous use of bone specific therapy: ever use, generic name of drug, date of first and last use and total number of years of use. Previous or current participation in clinical trials should be recorded and include the date of the trial(s) and treatment(s) tested should be documented.

Assessment of severity of pain should include a VAS 0–10 and Brief Pain Inventory [13] for adults or Wong Baker Facies [14] for children. The presence of night pain should raise a red flag for possible complications such as imminent fracture, bleeding into a cyst or very rarely malignant transformation. A potential neuropathic character to pain should be assessed using the PainDetect questionnaire [15]. The contribution of pain from surgical scars, referred pain from adjacent joints, local tendinosis, chronic pain disorders/ fibromyalgia should also be evaluated.

In the presence of focal and/or acute onset pain, acute or impending fracture, aneurysmal bone cyst or stress fracture especially in a deformed long bone should be considered. Mechanical pain can be provoked using the rotational stress test, for instance in lesions of the proximal femur. The FABER test: motion hip in Flexion, ABduction and External Rotation, is also recommended. Although very rare, sarcomatous change should be considered in the presence of diffuse and/or chronic pain, especially if progressive and unrelenting and also present at night. This should be further evaluated using CT/ MR imaging and discussed with the local sarcoma team. Mechanical/ weight bearing bone pain can signal a stress or impending fracture. This should trigger consideration for correction of alignment, and/or consideration for the necessity of a surgical procedure, possibly involving the use of an intramedullary titanium nail or custom-made titanium angled blade plate, based on the ‘bridging the defect’ principles, to stabilize the involved bone.

Physical examination of the skeletal system should include gait, deformity including leg length discrepancy to inform potential complications, areas of tenderness and range of movement of adjacent joints and presence of spinal kyphosis and scoliosis [16–18].

Bone-related laboratory testing [19–22] should include a standard biochemistry screen of renal profile, total alkaline phosphatase, bicarbonate, albumin-adjusted serum calcium, phosphate (see below), 25OH-vitamin D and parathyroid hormone.

Abnormal phosphate homeostasis, specifically renal phosphate wasting leading to hypophosphatemia, is important to diagnose as it is an important predictor of future fracture risk, as well as other complications [20, 22, 23]. In all subjects with suspected polyostotic disease, baseline overnight fasting phosphate levels should be checked. In case of use of phosphate supplements, serum phosphate should be measured whilst the patient is off supplements for at least a day. Values should be related to age specific reference ranges. Phosphate homeostasis is ideally assessed in the fasting state by concomitantly collecting serum phosphate and creatinine and second void urine phosphate and creatinine. This will enable calculation of the tubular reabsorption of phosphate (TmP/GFR) and calculated values should again be related to age-related reference ranges [http://members.iinet.net.au/~bill/java/tmp_gfr.html].

It is important to exclude other causes of renal phosphate wasting such as hyperparathyroidism and renal tubular acidosis. This can usually be done through history, examination and biochemical assessment e.g. dipstick urinalysis for glycosuria, measurement of serum bicarbonate and urinary amino acids. It should be noted that hypophosphataemia may *be episodic and assessment may need to be repeated if skeletal symptoms change and during linear growth.* If serum FGF-23 is to be measured the blood sample should be collected at least 7 days off phosphate/ vitamin D supplements, using an accredited assay/ laboratory if available.

Total alkaline phosphatase (ALP) is the minimum recommended biomarker for bone turnover. Other bone turnover markers are optional and include bone-specific ALP (with age related reference ranges), procollagen Type 1 N-terminal propeptide (PINP), C-terminal telopeptide (CTX-I). If these are not available, consider storing serum at − 20 °C for later analysis.

Skeletal imaging is the investigation of choice to determine skeletal burden depending on the clinical presentation [24–26]. All skeletal burden is detectable by age 15 years and clinically significant lesions by the age of 5 years [24]. To evaluate the extent of FD, whole body imaging using bone scintigraphy, whole body MR or low-dose 2D/3D radiography (e.g. EOS), to determine the presence and extent of skeletal involvement should be considered for all patients ≥ age 5 years. It should be noted that skull base lesions are likely to be missed by the EOS. Due to the possibility of false negative results, whole body imaging should be delayed in asymptomatic children until age 5 [24] and when the child can tolerate an MRI without general anaesthesia. Whole body or targeted skeletal imaging prior to age 5 should be considered when the benefits of early diagnosis outweigh the risks of waiting till the child is older. Scoring of the skeletal burden should be performed using the Collins’ validated method [25].

Areas of clinically significant axial and appendicular FD identified on bone scintigraphy should be imaged with conventional radiographs in two planes of the whole bone. A localized fine cut CT scan is preferred in case of clinical evidence of nerve entrapment.

#### Specific recommendations for spinal FD (Fig. 3c)

Evidence of scoliosis on physical examination should be confirmed with conventional radiographs. Once established, progression of scoliosis should be assessed with regular, periodic radiographs and pulmonary function testing. The periodicity of these examinations should be adjusted based on the severity and rate of progression, or lack thereof, in a given individual. Early consultation with spinal team and therapists is recommended and surgical fixation should be considered if Cobb angle is above 30 degrees depending on the rate of progression and location of the curve. [18, 27, 28].

#### Craniofacial FD (Flow chart Craniofacial FD)

The aim of staging cranio-facial lesions is to define and record the extent, distribution and impact of FD in the craniofacial skeleton. Following a history and physical examination the following tests are recommended where clinically relevant: objective assessment of facial asymmetry using clinical photography and 3D photography and assessment of psychological impact including using the Craniofacial Experience Index [29]. Radiological assessment includes standard radiological facial and orthognathic series and fine cut CT 1 mm or less slice thickness. If craniofacial lesions are adjacent to relevant structures or nerve pathways, referrals should be considered to the following specialties: craniofacial surgery, plastic surgery, ophthalmology, ENT & audiology, maxillofacial surgery and neurosurgery. A referral to a specialised craniofacial service should be considered if there is evidence of nerve impairment of functional impairment.

#### Dental FD

In case of dental involvement, panoramic radiographs and intraoral (periapical and bitewing) radiographs will provide assessment of both arches, as well as adjacent anatomic structures including maxillary sinuses, nasal cavity, mental foramina and mandibular canals. In addition, useful information will be provided regarding the presence of carious lesions, periodontal disease, or periapical disease, all important risk factors for osteonecrosis of the jaw (ONJ). More advanced imaging techniques include the use of cone beam computerized tomography (CBCT) assessing cortical and cancellous architecture with lower radiation exposure, magnetic resonance imaging (MRI), ^99m^Tc-methylene diphosphonate bone scintigraphy, and positron emission tomography, (PET).

### Extra skeletal system

#### Endocrine system

##### Ovarian assessment

(See Additional file 4: Flowchart Endocrine management: Gonadal Evaluation in Girls)

A targeted history and physical examination including history of breast development, vaginal bleeding and/or signs of estrogenization (e.g. below age 8 years), ovarian cysts (Fig. 2a), and irregular menses as defined as menstrual cycles that are shorter than 21 days or longer than 35 days).

All children should have a review of their growth curve for linear growth acceleration or deceleration and a standardised bone age examination [30]. If symptomatic, girls should have a random blood FSH, LH, estradiol and pelvic ultrasound.

##### Testicular assessment

(See Additional file 4: Flowchart Endocrine management: Gonadal Evaluation in Boys and Men)

A targeted history including history of pubertal development, and physical examination including Tanner staging including testicular volume [31, 32]. All males should have a testicular ultrasound at baseline (Fig. 2b) and after age 5 to characterise subclinical involvement consistent with MAS. If symptomatic, boys should have measurements of FSH, LH and free testosterone.

##### Thyroid assessment

(See Additional file 4: Flowchart Endocrine management: Thyroid Evaluation)

All patients should have a targeted history and physical examination, measurement of TSH, free T4 & total or free T3 and thyroid ultrasound to characterise subclinical involvement consistent with MAS (Fig. 2c) [33, 34]. Of note, in FD/MAS, hyperthyroidism is a T3 driven disease due to increased deiodinase activity [33] so that measurement of T3/T4 ratios is helpful, with a ratio of > 20 being indicative of disease.

##### Pituitary assessment

(See Additional file 4: Flowchart Endocrine management: Growth Hormone Excess Evaluation)

All patients should have a targeted history and physical examination including height measurement and comparison with mid-parental height [35, 36]. All children should have a review of their growth curve in relation to age and stage of pubertal development and head circumference SDS. Evaluation of growth velocity may be confounded by bone disease and/or additional endocrinopathies. All children should have their bone age evaluated, with determination of predicted adult height and comparison with Tanner stage and mid-parental height (e.g. Bayley N & Pinneau SR [37]. Evaluation of bone age may be confounded by bone disease.

All patients should have a random blood test for IGF-1, growth hormone (GH) and prolactin measurements. In children biochemical testing, especially of serum GH / IGF-1, may be misleading in the presence of precocious (or normal) puberty as likely to be well outside the normal age-related range. If there is a laboratory abnormality or clinical concern regarding GH-excess, the recommendation is to investigate further by measuring IGF-1. Such cases may require a glucose tolerance test and/or overnight growth hormone sampling to confirm the diagnosis. Pituitary MRI is indicated in case of abnormal biochemistry (Fig. 2d), although a normal pituitary MRI does not rule out the possibility of GH excess as the affected tissue may not be detectable by MRI [36].

Patients with endocrinopathies should comply with additional disease-specific screening programmes as per published guidelines, e.g. acromegaly and screening for colonic neoplasia [38].

##### Adrenal assessment

(See Additional file 4: Flowchart Endocrine management: Adrenal Evaluation (children))

Hypercortisolism presents exclusively in the first year of life and may spontaneously resolve [39, 40]. A targeted history and physical examination should be performed, to include a history of infantile illness, developmental delay, poor linear growth with excessive weight gain. In case of clinical suspicion of current hypercortisolism, 24-h urinary free cortisol, low dose dexamethasone suppression test, diurnal cortisol and adrenal CT should be performed. It is of note that adrenal involvement may present as adrenal insufficiency in later life reflecting previous (resolved) hypercortisolism. The ACTH stimulation test should be performed in all patients with a known or suspected history of neonatal hypercortisolism.

#### Dermatological lesions (Fig. 1)

A physical examination should be performed in all patients for typical café-au-lait macules (any size and darkness with characteristic jagged borders (Coast of Maine) [41, 42] Figure. The distribution typically respects the midline of the body). In adults, brown macular lesions may develop on the lips. Extra care should be taken to identify lesions in patients with darker skins.

### Evaluation of quality of life in FD/MAS

The evaluation of quality of life in patients with FD/MAS should be performed with language specific versions of the EQ5D-5 L [43] and SF 36 [44] in adults and the PEDS-QL [45] in children. Additional measures of anxiety and depression, e.g. using the Hospital Anxiety and Depression scale [46] and sleep, e.g. using the Epworth Sleepiness Scale [47] or Pittsburgh Sleep Quality Index [48] could be considered. In CFFD assessment of psychological impact including using the Craniofacial Experience Index [29] could be considered.

## Management of FD/MAS

### General measures

#### Provision of information about the disease

Provision of sufficient information about the disease to the patient and families is of outmost importance for this rare disease, which may be associated with debilitating manifestations, and for which there is no cure and no approved treatment. The aim is to empower patients and support them to develop to the best of their abilities.

Patients and their families should be informed of the non-inherited genetic nature of disease and that while malignant transformation can very rarely occur, FD/MAS lesions are almost invariably benign. They should also be informed that there are no known exposures that cause FD/MAS. Patients and their families should be given written information material about FD/ MAS and informed of the local regional / national / international patient groups including those based on social media for additional support. Patients should also be given details of “Expert” patients and specialist clinical centres / networks (e.g. European Reference Networks). Given the gaps in our knowledge of FD/MAS, research is high priority and patients should be given information about local research studies or trials.

##### Lifestyle advice

Advice should be given to optimize lifestyle factors which are associated with optimal bone health. Patients should be advised to achieve appropriate dietary calcium intake per age and achieve sufficient 25-OH vitamin D levels as per national guidelines, especially if pharmacological treatment with anti-resorptives is contemplated. Smoking cessation, alcohol moderation to < 3 units/ day and maintaining healthy weight should be discussed as required. Appropriate, safe and sufficient physical exercise to optimize fitness should be recommended with referral for physical therapy as required. Regular dental examinations should be recommended according to national guidance including control before starting medication. Patients should be advised about optimizing oral health to reduce the risk of oral infection. Educational materials, occupational advice and information on sexual health should be available and, where appropriate, how to access additional support. Consideration should be made for specific referral to a psychologist for those with moderate to severe disease, especially in the presence of significant physical disability and/or craniofacial impact. Referral to a social worker may also be required.

#### Exercise and rehabilitation

Advice should be given about appropriate physical exercise, to optimize cardiovascular fitness, and enhance educational and occupational performance [16, 17]. Rehabilitation and physical therapy may also be recommended to optimize strength and function, and attenuate loss of mobility. Orthopaedic review and orthoses maybe needed to correct any discrepancy in leg length (see below)

### Specific measures

#### Management of FGF-23 induced renal phosphate wasting

Patients with FGF-23-mediated hypophosphatemia, especially children, need to be referred to a metabolic bone specialist with experience in phosphate wasting disorders. Interpretation of serum phosphate levels is based on age. In adults and children, hypophosphatemia is associated with a higher risk of fractures and should be treated [20]. Having established that hypophosphatemia is due to FD-related FGF-23-induced renal phosphate wasting, treatment is similar to other disorders of FGF-23 excess. A baseline blood sample to measure PTH, serum adjusted calcium and eGFR, fasting urine for measurement of urinary calcium/creatinine ratio and renal ultrasound to establish pre-treatment status regarding possible nephrocalcinosis/nephrolithiasis should be performed. Treatment should be started with an active metabolite or analogue of vitamin D. This could be calcitriol (EU + US) 1μg/day in divided doses or alfacalcidol (only EU) 1.5μg/day in a single daily dose in adults and 15–60 ng/kg/d, divided bid for children. Doses may be increased as necessary, providing serum and fasting calcium / creatinine sample or 24-h urinary calcium measurments are regularly monitored to avoid hypercalciuria and the associated risk of nephrocalcinosis/ nephrolithiasis. The dose of active vitamin D should be titrated to suppress hyperparathyroidism and maintain the urinary calcium excretion just below the upper limit of the normal laboratory reference range. An ultrasound of the kidneys is recommended in case of persistent hypercalciuria or every year if the patient is on active vitamin D and phosphate supplements.

Phosphate supplements should be given in the form of a drink containing 1 mmol/ml of phosphate divided in multiple doses throughout the day e.g. 5-10 ml tds for adults and 1–3 ml/kg body weight qds for children. Phosphorus supplementation can also be expressed in mg/kg, especially during paediatric age, with a range from 15 to 60 mg/kg/day, divided in 4–5 doses. The dose of phosphate supplement should be titrated to maintain serum phosphate at the lower end or just below the normal laboratory reference range for serum phosphate. Care should be taken to avoid overtreatment. Patients should be advised of the potential for gastrointestinal upset and to consider taking a smaller dose more often.

Long-term phosphate supplementation is associated with chronic stimulation of parathyroid hormone secretion, potentially leading to 4-gland hyperplasia and autonomous hyperparathyroidism precluding the further use of active metabolites of vitamin D and requiring surgical intervention to remove the hyperplastic glands. PTH concentrations need to be monitored after one month of therapy and as frequently thereafter as required by dose changes and level of PTH while under treatment with phosphate.

#### Management of Scoliosis

Patients with scoliosis should be regularly monitored for progression. Early consultation with spinal team and therapists is recommended and surgical fixation should be considered if Cobb angle is greater than 30 degrees, depending on the rate of progression and location of the curve [18, 27, 28].

#### Management of bone pain (See Additional file 3: Flowchart Management of Bone Pain)

The strategy is to induce symptom remission [22, 49–57]. Key assessment tools for bone pain in FD are outlined above. The presence of night pain is red flag and the patient should be evaluated for complications including imminent fracture, bleeding into a cyst and malignant transformation. The presence of focal and/or acute onset pain may also indicate an acute or impending fracture (especially in a deformed long bone) or an aneurysmal bone cyst. Mechanical/ weight bearing bone pain can also signal a stress or impending fracture. The presence of a stress fracture should trigger consideration for correction of alignment, and/or consideration for the necessity of a surgical procedure, possibly involving the use of an intramedullary titanium nail or of a custom-made titanium angled blade plate, to stabilize the bone to prevent an uncontrolled fracture. (See Additional file 5: Flowchart Surgical management of FD of the proximal femur)

##### Pharmacological management

The first step in the pharmacological management of FD is to ensure supplementation (not correction) of hypophosphatemia if present (see above), and vitamin D repletion (according to national guidelines). For analgesics, consider paracetamol/acetaminophen as first line, followed by NSAIDs, if benefits outweigh cardiovascular, renal and gastro-intestinal risk. Bone therapies should be tried before recommending tramadol and other opioids. If neuropathic features such as burning or stabbing pain are present, consider an atypical analgesic ladder, e.g. amitriptyline, gabapentin, pregabalin and duloxetine. Of note, these medications are proposed by analogy with other painful diseases, but no specific trial has been conducted in FD. If pain management becomes complex, consider pain specialist review. Consider cognitive therapies and sleep hygiene interventions as used for chronic pain. Referral for physical therapy is recommended to optimize function and reduce pain.

Bisphosphonates are proposed for persistent, moderate to severe pain as defined by VAS score of > 3/10. It remains unclear whether bisphosphonates reduce FD lesion size or progression in children or adults. Their ability to increase local bone density or preventing complications has not been established. Prior to their use, ensure that the patient is normo-calcaemic, has an adequate dietary calcium intake and an adequate 25OH vitamin D level (according to national guidelines) and that the creatinine clearance is ≥35 ml/min. Importantly, hypophosphatemia should be corrected as best a possible for least 6 months prior to initiating bisphosphonates. Treatment protocols for the most commonly used bisphosphonates are shown in the Table 3. There is no evidence to support use of alendronate or risedronate for treatment of pain in fibrous dysplasia. High dose alendronate, 40 mg daily, does not improve pain in a controlled trial [53]. Oral bisphosphonates, at any dose, are therefore not recommended for the treatment of bone pain.

**Table 3:** Example of intravenous bisphosphonate regimens used for the management of bone pain in patients with fibrous dysplasia / McCune Albright Syndrome

	Pamidronate			Zoledronate		
	Loading Dose	Stop if	Comments	Loading Dose	Stop if	Comments
Paediatric^a^	1 mg/kg × 2 on consecutive days up to a total of 90 mg. If no response, consider repeat at week 8.	No response after 2 cycles	Consider use of 20–30 ng/kg/day calcitriol or 30–50 ng/kg/day alfacalcidol up to 1 mcg/day and calcium supplements for 4 days after first injection to maintain calcium homeostasis especially in those with high disease burden.	0.025 mg/kg. If no response, 0.025 mg/kg at week 8. If still no response, 0.05 mg/kg at week 24	No response after 3 doses	Consider use of 20–30 ng/kg/day calcitriol or 30–50 ng/kg/day alfacalcidol up to 1 mcg/day and calcium supplements for 4 days after first injection to maintain calcium homeostasis especially in those with high disease burden
Adult	90 mg ×2 consecutive days every 6 months	No response after 2 cycles		Up to 5 mg monthly (switch to maintenance dose when achieve response)	No improvement in pain after 3 doses	

Legend: ^a^ Dosing examples are intended for children age 3 and above. If pain management is needed for a child under age 3, recommend consulting with a paediatric metabolic bone specialist. ^b^Monthly zoledronate is usually reserved for severe cases with a very high skeletal burden. Most cases require annual or bi-annual therapy

Intravenously administered pamidronate and zoledronate can be equally considered (Table 3). The aim of initial dosing is to establish whether bisphosphonates are effective in providing pain relief. Several doses may be required initially to establish whether they are effective in providing analgesia. Subsequent dosing intervals should be determined according to need for analgesia and response to previous doses. In general, one should aim to increase the interval between doses over time.

Patients and families should be counselled about short and long-term risks and concerns possibly associated with the use of bisphosphonates. Consideration should be given to appropriate monitoring of bone health according to dose and duration of therapy which may include the serum measurement of bone turnover markers and bone density. Dental evaluation is recommended prior to treatment to minimize the risk of osteonecrosis of the jaw. There have been no reports of atypical femur fractures despite the high cumulative doses used long-term in some of the reported large case series [52].

If there is an inadequate clinical improvement, as measured by no or insufficient change in pain score, other non-bone causes of pain should be excluded before switching to the other parenteral bisphosphonate. If there is still no improvement in pain, then do not continue with bisphosphonate therapy and review other causes of pain and consider other analgesic strategies.

Evidence for the dosage, efficacy and safety of other anti-resorptive agents such as denosumab is currently scarce and the use of this agent is not recommended outside specialist centres, preferably in the context of a clinical study or trial. The main concern raised so far with the use of this agent in FD is the apparent increased risk of significant hypercalcaemia following cessation of therapy in children [58] and rebound increase in fracture rate in adults treated with denosumab for osteoporosis when therapy is discontinued [59].

#### Management of Mazabraud syndrome

More often than not myxomas of Mazabraud’s syndrome are asymptomatic, and require no intervention. However, surgical excision is recommended if they become painful,. There may be a local recurrence risk of up to 25%. Follow-up scanning with MRI is dependent on clinical symptoms. Longer term surveillance and testing for other extra-skeletal manifestations is recommended in polyostotic FD and in the presence of other features of MAS.

#### Management of Endocrinopathies

##### Ovarian pathology [52–57]

(See Additional file 4: Flowchart Endocrine: Management of precocious puberty in girls*)*

In general, ovarian surgery for cysts should be avoided, as disease is usually bilateral. Ovariectomy should only be performed when there is a risk of torsion and after expert consensus. Patients should be informed that the risk of torsion is small.

Treatment for precocious puberty is indicated if bone age is advanced and there is frequent bleeding. Psychological distress and the patient’s age need to be taken into account as the height outcome is only improved in those <6ys at onset i.e. the very young group. First line therapy is letrozole, with tamoxifen or fulvestrant as second line or adjuvants. Patients should be monitored for central puberty and the need to add a gonadotropin-releasing hormone analogue (GnRHa), e.g. leuprolide.

Adult women should be monitored for dysfunctional uterine bleeding. For contraception and HRT it may be prudent to avoid additional estrogenic compounds to avoid a possible increase in the risk for breast cancer, since patients with MAS may be at an increased risk of estrogen positive breast cancer [60], and patients with precocious puberty have both longer exposure as well as continued intermittent autonomous production of high levels of estrogen up until the menopause.

##### Testicular pathology [31]

(See Additional file 4: Flowchart Endocrine: Management of precocious puberty in boys & men)

In general, surgery should be avoided. Structural lesions are rarely of clinical significance. Treatment for precocious puberty is indicated in case of an associated elevated serum testosterone and/or bone age advancement. Combination of testosterone receptor blocker and aromatase inhibitor are needed as well as monitoring for central precious puberty, in which case GnRHa may need to be added.

Testicular lesions should be examined annually and males informed to perform self-examinations. Annual ultrasonography is indicated for palpable lesions or for lesions causing an overall increase in the size of the testes (relative to other testis or stage of puberty).). In adulthood no routine ultrasounds are advised, unless lesions are changing. Consider biopsy for lesions that are changing in size.

##### Thyroid pathology [33, 34, 61]

(See Additional file 4: Flowchart Endocrine: Management of hyperthyroidism)

In the short-term, carbimazole or methimazole are recommended for hyperthyroidism, whereas thyroidectomy or radio-ablation are recommended for long standing hyperthyroidism of more than 5 years.. Patients can be treated with I-131 but considering the evaluation of thyroid nodules one should perform full evaluation of the nodule before treating with I-131.

Annual long-term monitoring is advised due to the possibility of regrowth. For, children aged less than 10 years with an abnormal US and normal thyroid function tests (TFTs), physical examination, growth velocity, and TFT’s should be monitored every 6 to 12 month. In case lesions are found, follow up of patients with FD/MAS-related thyroid disease should be performed according to current (inter) national guidelines [62–64].

##### Growth hormone excess [36, 65–69]

(See Additional file 4: Flowchart Endocrine: Management of growth hormone excess)

Somatostatin analogues are first line therapies with second line options including pegvisomant, alone or in combination with octreotide or lanreotide at the discretion of the treating physician. Pituitary surgery is recommended for patients resistant to medical therapy. Total hypophysectomy is required as the whole gland is usually involved and removing just the adenoma is not enough to control the excess production of growth hormone. Surgery is almost universally complicated by co-existent craniofacial FD, and so always challenging. Maximal medical therapy is standard of care, and pituitary radiation should be a final recourse due to the risk of malignant transformation of skull base FD [36, 70]. The treatment goals are to achieve an IGF-1 Z-score between − 2 and + 1. Treatment should be monitored by annual growth velocity, head circumference, and IGF-1 in all growing children. Assessment of additional pituitary hormone deficiencies is recommended after hypophysectomy and/or radiation therapy.

##### Adrenal pathology [39, 40]

(See Additional file 4: Flowchart Endocrine: Management of hypercorticolism)

Metyrapone is the preferred first-line agent with etomidate for critically ill patients. Other options include mitotane and ketoconazole. Ketoconazole should be used with caution as it is frequently associated with hepatic toxicity. However, bilateral adrenalectomy is usually required. Unilateral adrenalectomy may be considered in stable patients who have the appearance of unilateral disease. Of note, spontaneous resolution occurs in up to 1/3 of patients. In stable patients, adrenalectomy could be deferred with close monitoring for resolution. The benefits and risks of medical therapy should be balanced with the potential developmental risks of continued hypercortisolism. Assessment of adrenal insufficiency is recommended after resolution of hypercortisolism.

#### Management of other extraskeletal manifestations of FD/MAS

##### Haematological manifestations [71]

Platelet function activation testing should be performed if there is a history of bleeding. If abnormal, this can be corrected pre-operatively by platelet transfusion.

##### Gastrointestinal manifestations [72–75]

Gastrointestinal polyps and hepatobiliary neoplasms have been reported in FD, although their clinical significance is unclear. Pancreatic disease, including intraductal papillary mucinous neoplasms, have also been described and a single case of malignant transformation has been reported. Pancreatic pathology may be associated with acute or chronic pancreatitis and serum amylase should be measured if there is history of abdominal pain. It is recommended that all patients are evaluated for gastrointestinal symptoms and imaging considered for symptomatic patients and those with a history of pancreatitis.

##### Malignancies [4, 31, 61, 76–79]

There is a likely small increased baseline risk of developing malignancies in mutation-bearing tissue as well as in lesions with high turnover. Patients should be encouraged to be compliant with existing cancer screening programmes for the general population such as screening for breast and prostate cancer, as an increased risk for these malignancies has been observed in patients with FD. Patients with endocrinopathies should comply with additional disease-specific screening programmes as per published guidelines, e.g. acromegaly and screening for colonic neoplasia [38].

Patients should be advised to avoid additional risk factors (excessive radiation exposure, smoking, excessive alcohol, etc.)

#### Surgical management of FD/MAS

Management of orthopaedic issues requires working within a multidisciplinary team to ensure optimal phosphate status and exclusion of endocrine abnormalities that exacerbate skeletal disease (e.g. GH excess and T3 thyrotoxicosis). Review by a specialist orthopaedic surgeon is needed for fracture, potential mechanical/ tumour bone pain or limb deformity. Limb deformity requires early assessment for prophylactic surgery to prevent worsening deformity, pain and fracture [80]. Leg length discrepancy requires assessment for the need for orthotics and corrective surgery.

The presence of joint-based pain may require referral for physiotherapy, analgesics, osteotomy (especially if there is deformity) and/or arthroplasty. Whereas curettage may be effective in a very low volume bone lesion, curettage filled with bone (auto or allogenic) grafts is not recommended as it is ineffective and may be associated with complications. In general, external fixation is only used for temporary correction and/or fixation, while waiting for a more definitive custom-made printed implant. Preferred internal fixation is with a titanium intramedullary nail, bridging the involved bone where possible. In special circumstances plate fixation can be considered. Internal fixation using conventional titanium plates or custom-made titanium plates by bridging the involved bone is another option. There appears to be a higher rate of fracture after steel plating vs. titanium plating and this may be related to the better elastic modulus of titanium compared with steel. The stabilization procedure is often facilitated by performing a correction osteotomy. Allogeneic cortical strut grafting (tibia or fibula) has been used for bridging the involved bone, for small FD lesions, but is not recommended after incomplete or complete fractures.

Given that FD lesions are vascular, blood loss can be significant and staged procedures are therefore recommended if multiple surgeries are planned in order to minimize the need for transfusions. Consider interventional radiological control with either embolization or balloon catheter for very high flow lesions. There is so far no evidence for added value of using of bisphosphonates to decrease vascularity of FD lesions pre-operatively although this warrants testing formally in future studies. For children or severely affected adults, active follow-up is needed in the medium and long term as the deformity may recur and require further surgery. Rehabilitation including physiotherapy, hydrotherapy and mobility aids should be available as needed after surgery.

Also see: (See Additional file 5: Flowchart Surgical management of FD of the proximal femur)

#### Management of Craniofacial FD (CFFD) [51, 81–86] (See Additional file 6: Flowchart: Management of CFFD)

FD of the craniofacial skeleton is variable in its behaviour and the multidisciplinary team caring for patients with CFFD needs its combined expertise to cater for all treatment options. Any planned surgical treatment should be carefully coordinated with other specialists involved in the patient’s care. Working within a multidisciplinary team, thus ensures among other aspects of management, optimal phosphate status, adequate vitamin D and preoperative correction of endocrine abnormalities, such as GH excess and T3 thyrotoxicosis, that may exacerbate skeletal disease. The balance of risks and benefits of extensive resection and/or reconstruction needs to be carefully outlined in great detail in patients with CFFD. Active watch and wait policies are often the preferred management strategy, as long-term outcomes in terms of regrowth and pain are very variable and generally poorly predictable.

If CFFD is identified at baseline or at subsequent monitoring evaluations, the patient should be referred for a formal assessment to a craniofacial service with experience in the care of patients with CFFD. The goals of treatment are: a) Prevention of functional loss – especially hearing and vision; b) Arrest or reduction of physical disfigurement; c) Prevention of secondary deformity; d) Minimisation of long-term morbidity from CFFD and its treatment.

The structure of the individualised package of care of CFFD is based on the extent of craniofacial involvement and on the following concepts. If possible, care is to be provided locally, but any decisions regarding surgical intervention should be taken by a multidisciplinary specialised team including physicians and surgeons with experience in managing CFFD. Scheduling of periodic evaluations should be organised by the central coordinating CF team with CFFD patients being reviewed at least annually or more frequently depending on the extent of their disease and risk of complications. Baseline and periodic CT scans of the head should be performed in children, usually every 2 years or less frequently based on the localisation and severity of the lesion(s). Regular imaging is not indicated In adults, and timing of the scans should be based on symptoms, at most every 5 years in those without symptoms.

Although the primary aim of treatment should always be to preserve function, treatment of primary deformity and prevention of secondary deformity are also important. Advanced imaging techniques and 3-dimensional analysis of scans together with virtual surgical planning and computer-aided manufacturing and design of patient-specific implants should be regarded as the standard of care in surgery of FD of the craniofacial skeleton. Simple curettage is not recommended as it is ineffective and may increase the risk of complications.

##### Specific CFFD management recommendations

Lesions of the cranial vault usually present as a mass, asymmetry or other form of physical deformity and treatment options include: burring of the lesion to reduce bulk and achieve symmetry; subtotal excision and reconstruction; complete excision of the lesion and reconstruction of the calvarial defect.

Lesions of the skull base should be monitored by periodic evaluation by the craniofacial team. Any evidence of functional embarrassment of the structures exiting the skull base should prompt a review by a skull base surgeon. Surgery should be avoided in the absence of functional deficits. An assessment of hearing should be performed annually in all patients with skull base disease.

Lesions of the frontal bone usually present as a physical deformity or asymmetry. These lesions often alter orbital morphology, affect the position of the globe and can cause significant deformity. Although diplopia is not a common symptom of CFFD, surgical intervention may be associated with this disabling complication. Pre-operative ophthalmological evaluation is essential to establish the likelihood of post-operative diplopia and the fusion range. Surgical options include: burring of the lesion to reduce bulk and achieve symmetry; subtotal excision and reconstruction; excision of CFFD lesion and reconstruction of the fronto-orbital defect and correction of the globe’s position. Prophylactic optic nerve decompression is not recommended. Proven visual deterioration with sequential ophthalmological evaluation warrants an urgent evaluation by a craniofacial surgeon with experience in the management of fibrous dysplasia.

Lesions of the naso-ethmoid region can affect the airway and globe of the eye’s position. An ENT evaluation is recommended in addition to a detailed ophthalmological evaluation. Treatment strategies are aimed at: reducing airway obstruction; correcting globe position and visual function and correcting physical deformity. Surgical options include: subtotal excision via limited access/ endo-nasal approach and radical excision with reconstruction of the skull base and orbits.

Maxillary lesions affect both orbital morphology and contents as well as dental occlusion. Surgical strategies include: preserving occlusal function and dentition (including tooth buds as well as erupted teeth); correcting globe position and visual function; reducing secondary deformity and using stealth incisions to minimise surgical morbidity. Surgical options include: burring of lesions to achieve symmetry and reduce bulk; subtotal excision and reconstruction of orbital floor and maxilla as required; radical excision and reconstruction of the orbit and maxillary arch to enable dental rehabilitation.

Mandibular lesions: Although CFFD of this site often presents with a mass in the lower border of the mandible, disease progression will lead to dysfunction. Like the maxilla, surgical strategies should be directed at: preserving occlusal function and dentition (including tooth buds as well as erupted teeth), reducing secondary deformity; and using stealth incisions to minimise surgical morbidity. Surgical options include: burring of lesions to achieve symmetry and reduce bulk; subtotal mandibular excision and reconstruction; and radical excision and reconstruction.

##### Oral and dental management in FD/MAS [84, 87–89]

Patients with FD, including CFFD, do not require special dental management and are able to undergo routine dental and orthodontic treatments without exacerbating their craniofacial lesions. However, malocclusion, dental crowding and smoking contribute to poorer oral hygiene [82].

Dental anomalies such as oligodontia, enamel hypoplasia, dentin dysplasia, taurodontic pulp, odontoma, tooth displacement, malocclusion, and high caries activity have been reported in 28% of patients with craniofacial FD. For this reason, all patients with CFFD should be carefully monitored for the appearance of these dental anomalies during growth

The risk of ONJ is discussed in the section on oral bisphosphonates. Management of ONJ is based on the stage of the disease, size of the lesions, and the presence of contributing drug therapy and comorbidity [88]. An important preventative strategy includes maintenance of good oral hygiene, elimination or stabilization of oral disease prior to initiation of treatment with an anti-resorptive agent and as far as possible avoidance of invasive dental procedures during treatment. Frequent recalls may be required for scaling and root planning to control dental plaque accumulation. Orthodontic tooth movement tends to be rapid in jaws with fibrous dysplasia and relapse is more common as teeth tend to return to their original position after removal of orthodontic appliances due to poor quality of FD bone.

Orthodontic treatment must be preceded by radiological evaluation to detect remodeled areas to inform orthodontic therapy. Functional removable appliance therapy should be preferred whenever possible. Fixed appliance therapy requires the maintenance of excellent oral hygiene conditions. In the vast majority of cases of craniofacial FD, orthognatic surgery is not needed, and observation is the correct approach. Indications for surgery include documented progressive, severe pain, or severe disfigurement. Results have been shown to be stable with no recurrence after surgery in adults.

Orthognatic surgery helps to restore stable occlusion and good facial aesthetics, but should be avoided in growing patients as in young patients as abnormal facial growth has been described in young patients operated during the active phase of growth.

## Conclusion

These best practice guidelines have been developed by an international collaboration between multiple clinical specialities, patients and patient advocacy groups, using the best evidence available. The FD/MAS guidelines are intended to improve the clinical care of patients across the world by addressing diagnosis, staging, treatment and monitoring aspects of their care given the potential serious risks to patient outcomes with late diagnosis [90]. The provision of a Patient Checklist (See Additional file 1: Fibrous Dysplasia and McCune-Albright Syndrome: A Checklist of P atients and Doctors) is aimed at informing and empowering patients to seek excellence of healthcare for their disease. Describing standards across the clinical care pathway enables clinical services to be audited, helps in the identification of areas of the patient pathway that require service improvement and facilitates cross-border sharing of best clinical practice between clinical services in different countries. These guidelines have additionally highlighted important gaps in our knowledge about FD/MAS and raise the importance of implementing international registries and cohort studies with active collaboration of patients and families. Currently, such initiatives include the Fibrous Dysplasia Foundation Registry (https://fibrousdysplasia.org), RUDY study (www.rudystudy.org) [91], James Lind Alliance Priority Setting Partnership for Rare Musculoskeletal Diseases in Adulthood (http://www.jla.nihr.ac.uk/) and European Reference Networks for rare bone (http://ernbond.eu/) and endocrine diseases (https://endo-ern.eu). The FD/MAS consortium commits to developing an audit tool of key performance and experience measures to an international audit of practice and to reviewing these recommendations at least every 5 years to reflect new evidence in FD/MAS natural history and management.

## Additional files

Additional file 1: Fibrous Dysplasia and McCune-Albright Syndrome: A Checklist for Patients and Doctors. (DOCX 35 kb)

Additional file 2: Flowcharts Skeletal Evaluation. (PPTX 47 kb)

Additional file 3: Flowcharts Management of Bone Pain. (PPTX 45 kb)

Additional file 4: Flowcharts MAS Endo. (PPTX 64 kb)

Additional file 5: Flowcharts Surgical Management of Proximal Femur. (PDF 34 kb)

Additional file 6: Flowcharts Management of CFFD. (PPTX 36 kb)

## Data Availability

Not applicable.

## Abbreviations

CBCT: Cone Beam Computerized Tomography
CFFD: Craniofacial Fibrous Dysplasia
CT: Computerised Tomography
FD: Fibrous Dysplasia
FD/MAS: Fibrous Dysplasia / McCune Albright Syndrome
GDG: Guideline Development Group
MRI: Magnetic Resonance Imaging
NGS: Next generation Sequencing
ONJ: Osteonecrosis of the Jaw
PET: Positron Emission Tomography
