# Correction to: Best practice management guidelines for fibrous dysplasia/McCune-Albright syndrome: a consensus statement from the FD/MAS international consortium

**DOI:** 10.1186/s13023-019-1255-6

**Published:** 2019-11-21

**Authors:** Muhammad Kassim Javaid, Alison Boyce, Natasha Appelman-Dijkstra, Juling Ong, Patrizia Defabianis, Amaka Offiah, Paul Arundel, Nick Shaw, Valter Dal Pos, Ann Underhil, Deanna Portero, Lisa Heral, Anne-Marie Heegaard, Laura Masi, Fergal Monsell, Robert Stanton, Pieter Durk Sander Dijkstra, Maria Luisa Brandi, Roland Chapurlat, Neveen Agnes Therese Hamdy, Michael Terrence Collins

**Affiliations:** 1 Nuffield Department of Orthopaedics, Rheumatology and Musculoskeletal Sciences, University of Oxford, Oxford, UK; 2 Skeletal Disorders and Mineral Homeostasis Section, National Institute of Dental and Craniofacial Research, Bethesda, MD USA; 3 Department of Medicine, Division of Endocrinology & Center for Bone Quality, Leiden University Medical Center, Leiden, The Netherlands; 4 Department of Plastic Surgery, Craniofacial Centre, Great Ormond Street Hospital for Children NHS Trust, London, UK; 5 Section of Paediatric Dentistry, University of Turin, Turin, Italy; 6 Department of Oncology & Metabolism, University of Sheffield, Sheffield, UK; 7 Metabolic Bone Team, Sheffield Children’s Hospital, Sheffield, UK; 8 Endocrine Department, Birmingham Women’s and Children’s NHS Foundation Trust, Birmingham, UK; 9 European Association of Friends of McCune-Albright Syndrome (TO), Turino, Italy; 10 Fibrous Dysplasia Support Society, Birmingham, UK; 11 Fibrous Dysplasia Foundation, Grandville, USA; 12 Department of Drug Design and Pharmacology, University of Copenhagen, Copenhagen, Denmark; 13 Department of Surgery and Translational Medicine, University of Florence, Florence, Italy; 14 Paediatric Orthopaedic and Trauma Surgery, University Hospitals Bristol NHS Foundation Trust, Bristol, UK; 15 Department of Orthopaedic Surgery, Nemours Children’s Hospital, Orlando, Florida USA; 16 Department of Orthopaedic Surgery, Leiden University Medical Center, Leiden, The Netherlands; 17 INSERM UMR 1033 and Université de Lyon, Lyon, France

**Correction to: Orphanet J Rare Dis (2019) 14:139**

**https://doi.org/10.1186/s13023-019-1102-9**

The original version of this article [1] unfortunately included an error to an author’s name. Paul Arundel was inadvertently presented as Paul Arunde.

The correct author name has been included in the author list of this Correction article and updated in the original article.
