# Supplementary material for: Best practice management guidelines for fibrous dysplasia/McCune-Albright syndrome: a consensus statement from the FD/MAS international consortium
Source: Orphanet J Rare Dis. 2019 Jun 13;14:139. doi: 10.1186/s13023-019-1102-9 (PMC6567644; doi:10.1186/s13023-019-1102-9)
Supplement: Supplementary file 1 — Fibrous Dysplasia and McCune-Albright Syndrome: A Checklist for Patients and Doctors. (DOCX 35 kb) [file 13023_2019_1102_MOESM1_ESM.docx]

| Fibrous Dysplasia and McCune-Albright Syndrome: A Checklist for Patients and Doctors |
| --- |
| A Tool from the FD/MAS International Consortium |

# About This Resource

Fibrous dysplasia/McCune-Albright syndrome (FD/MAS) is a rare and complicated disease that can affect the bones, skin and hormone glands.

FD/MAS is caused by a random genetic mutation. It is not inherited, and it cannot be passed down.

FD/MAS is so rare that you or your child could be the first person with FD/MAS that your doctors have ever treated. That means that your doctors might not be sure if FD/MAS is the right diagnosis or how to help you or your child.

The Clinical Pathway is a clear set of instructions for your doctors to follow. It was created by a team of top medical experts from across the globe who have treated hundreds of patients with FD/MAS and have learned from experience the best ways to take care of FD/MAS patients. These experienced doctors published this guide in 2018 because they want to help other doctors around the globe understand which tests and treatments they should order for patients with FD/MAS. You can print out copies of the Clinical Pathway and give one to every doctor who helps you manage FD/MAS symptoms.

Part of this Clinical Pathway is this checklist for patients and parents to help discussion with your doctor and healthcare team. We want this checklist to help you plan and get the most of your doctor appointments. There are three parts to the checklist:

Part 1. Questions you should ask about your diagnosis

Part 2. Questions you should ask to help plan your care

Part 3. Questions your doctor may ask you

Independent translations of this Checklist may be available in other languages. Please contact a national or international FD/MAS patient association for more information.

Asociación de Displasia Fibrosa, displasiafibrosa.es, Spain

Associação McCune-Albright Brasil, masfd.blog, Brazil

Association MAS-FD, masfd.org, France

European Association McCune-Albright Syndrome (EAMAS), eamas.net, Italy

Fibrous Dysplasia Foundation, fibrousdysplasia.org, USA

Fibrous Dysplasia Support Society, fdssuk.org.uk, United Kingdom

Patiëntenvereniging Fibreuze Dysplasie, fibreuzedysplasie.eu, Netherlands

# Questions to Ask About My Diagnosis

## Which parts of my body are affected by this disease?

| *Write down the answers here.* | Why is this important?  FD is usually present from the time you were born, and the bone disease is usually visible on bone scans by the age of 5. It can occur in almost any bone in the body but usually occurs in the head, legs and ribs. The same mutation that causes FD in the bone also can occur in skin, endocrine tissue (the glands in your body that make hormones) and other organs, so it’s important to check to see if those parts of your body are affected too. |
| --- | --- |

## Do I need more tests to find out if other parts of my body are affected?

| Blood and urine tests to look for problems with my:   - Ovaries - Testicles - Thyroid - Growth Hormone - Adrenal - Phosphate levels | Specialist eye sight screening  Hearing screening  Nuclear/isotope bone scan  CT scan  MRI scan  Ultrasound scan of the thyroid  Ultrasound scan of the testes  Tests to look at my pancreas  Tests to look at my gastrointestinal tract or gut | Why is this important?  All of these tests can help locate what areas of your body the disease affects, and how seriously they are affected.  Some of these tests can detect hormonal imbalances that can make your bone disease worse, unless they are treated correctly. |
| --- | --- | --- |

## Which of my test results were normal and which were abnormal?

| *Write down the answers here.* |
| --- |

## What type of fibrous dysplasia do I have?

| Monostotic fibrous dysplasia – my disease only affects one part of my skeleton  Polyostotic fibrous dysplasia – my disease affects more than one part of my skeleton  McCune-Albright syndrome – my disease affects more than one organ  Bone(s)  Skin, causing birthmarks and/or skin discoloration that are sometimes called café-au-lait spots  Glands, causing abnormal hormone levels  Muscle, causing lumps, which is also called Mazabraud’s syndrome | Why is this important?  In order to get the right types of medical monitoring, and to predict the success of different types of treatments, you and your doctor need to know what type of FD you have. |
| --- | --- |

## How was my diagnosis made?

| History and Physical exam, plus  X-rays  CT scan  Bone scan  Blood tests to look for hormonal or other problems  Biopsy/sample of tissue  Genetic tests  Other test: _____________________ | Why is this important?  If you receive the diagnosis of monostotic FD, it’s important to know that there are many diseases that may look like monostotic FD, that have very different treatment needs. Make sure that your radiologist and other doctors rule out these other diseases. They may suggest genetic testing from biopsies. Those tests can be helpful, but it’s important to know that people who have FD can still get negative results, even if they do have FD.  If you receive a diagnosis craniofacial or polyostotic FD it may be important to look for hormonal problems to confirm you don’t have McCune-Albright syndrome. |
| --- | --- |

# Questions to Help Plan My Care

## Can I have a copy of my test results, x-rays and scans, to keep?

| Why is this important?  Over time, you will probably see different doctors, and each of them may need to be able to access your full records in order to help you. It is important to note that many hospitals and clinics destroy medical records including test results, including x-rays, after a few years. It is good to have a back-up set of copies of all of your medical results.  Start collecting your records right away. |
| --- |

## How will my fibrous dysplasia/McCune-Albright syndrome be managed and monitored?

| What lifestyle advice can you give?  What kinds of exercise might be safe or helpful?  Should I take certain supplements? | If I have bone pain, what should I try to do to manage that pain?   - Non-medication options? - Medication options? - Surgical options? - What makes sense to try first? | Do I need phosphate treatment? | Do I need hormone treatments for my:   - Ovaries - Too much thyroid hormone - Too much growth hormone - Too much or too little adrenal activity |
| --- | --- | --- | --- |
| Should I receive psychological support for pain and/or psychosocial issues? | Should I receive physical therapy for pain and/or mobility? | Are there surgeries I may need? How will I know when surgery is a better option than “wait and see”? | Of the treatments available to me, what are the risks and benefits as compared to just monitoring the disease? |
| Why is this important?  While there aren’t any treatments known to stop or reverse the growth of FD, there are steps you can take to prevent fracture, stay mobile, treat pain, keep your hormone levels under control, and stop FD from interfering with the activities of daily life. | | | |

## How often do I need to see you, and repeat any tests that I’ve already had?

| *Write down the answers here.* | Why is this important?  Your schedule of visits and tests will depend on many things: your age, what type of FD you have, the location of your FD, the size of your FD, which organs other than bone are affected, and the results from your first set of hormone tests and other tests. |
| --- | --- |

## Do I need to see other specialists?

| Pediatrician  Endocrinologist  Rheumatologist  Orthopedist  Spinal surgeon  Craniofacial surgeon  Ophthalmologist or Neuro-Ophthalmologist | Maxillofacial surgeon/ Ear Nose Throat (ENT) doctor/ Otolaryngologist  Dental specialist  Neurosurgeon  Physical therapist  Psychologist  Pain Specialist | Why is this important?  FD/MAS affects so many systems that you might need multiple doctors, who each specialize in a different part of the body. Try and pick doctors who have experience with FD/MAS, who are willing to read new research on care (like this packet!), and who will work together to make recommendations and plans for your care. |
| --- | --- | --- |

## What information can you give me to help me make the best choices for my care?

| Is there written information I can have about fibrous dysplasia / McCune Albright using simple language so that I can learn more about it? | Which national or international patient group should I contact, such as the Fibrous Dysplasia Foundation, Fibrous Dysplasia Support Society UK, Associazione Europea amici della Sindrome di McCune-Albright, or Patiëntenvereniging Fibreuze Dysplasie? | Is there a medical center where I could receive care from a team of doctors who see many patients with fibrous dysplasia/McCune-Albright syndrome, and have lots of experience with patients like me? This center might be called a “center of excellence,” or a “reference network.” | Are there any research studies I can join? |
| --- | --- | --- | --- |

# Questions My Doctor May Ask Me

Your doctor may need to gather additional information to find out how your case of FD/MAS is affecting you.

It is important to note that the list below is very thorough, meaning you will probably never experience many of these symptoms on the list. For example, if the only place in your body where you have FD is in your leg then it would not be possible for FD to cause a problem with hearing of vision.

The list below is simply a way for you to begin to think about what symptoms (if any) you do have so that together, with your healthcare team, you can determine the best way to manage those symptoms.

## How has your FD/ MAS affected you?

| In what ways does FD/MAS affect  Your relationships:  at work or school  with family and friends  Your standard of overall health and happiness  Self esteem  Your ability to dress, wash, sleep and eat  Do you have a record of your height measurements? Did you grow normally/Are you growing normally?  What age did you hit puberty?  Have you broken any bones? If so which bones and when.  Are you able to walk without difficulty?  Do you have pain, tenderness, discomfort or numbness in your bones or joints? If yes then when did it start, what does it stop you doing, and what treatments have you tried.  Do you have a history of dental problems or surgeries?  Do you have problems with your eye sight or hearing?  What medications do you take now and what have you taken in the past?  Do you have any history of hormonal imbalances? | Why is this important?  .  The only way your doctor will become aware of what you’ve experienced, and what you’re experiencing is if you tell them or show them. It’s the first step to receiving help  For example, growing fast as a child, or hitting puberty early could be clear signs that you have McCune-Albright syndrome, and there may be need for additional treatments or monitoring.  Broken bones, difficulty walking, pain, dental problems or vision/hearing impairment could be signs of FD in that part of your body, or that the FD that is known to be in that part of your body needs additional treatments.  Dental problems could be a sign of FD in the jaw. Even if you don’t have FD in the jaw, a history of dental problems might also put you at risk of other complications.  . |
| --- | --- |
