# Supplementary material for: Best practice management guidelines for fibrous dysplasia/McCune-Albright syndrome: a consensus statement from the FD/MAS international consortium
Source: Orphanet J Rare Dis. 2019 Jun 13;14:139. doi: 10.1186/s13023-019-1102-9 (PMC6567644; doi:10.1186/s13023-019-1102-9)
Supplement: Supplementary file 2 — Flowcharts Skeletal Evaluation. (PPTX 47 kb) [file 13023_2019_1102_MOESM2_ESM.pptx]

## Slide 1
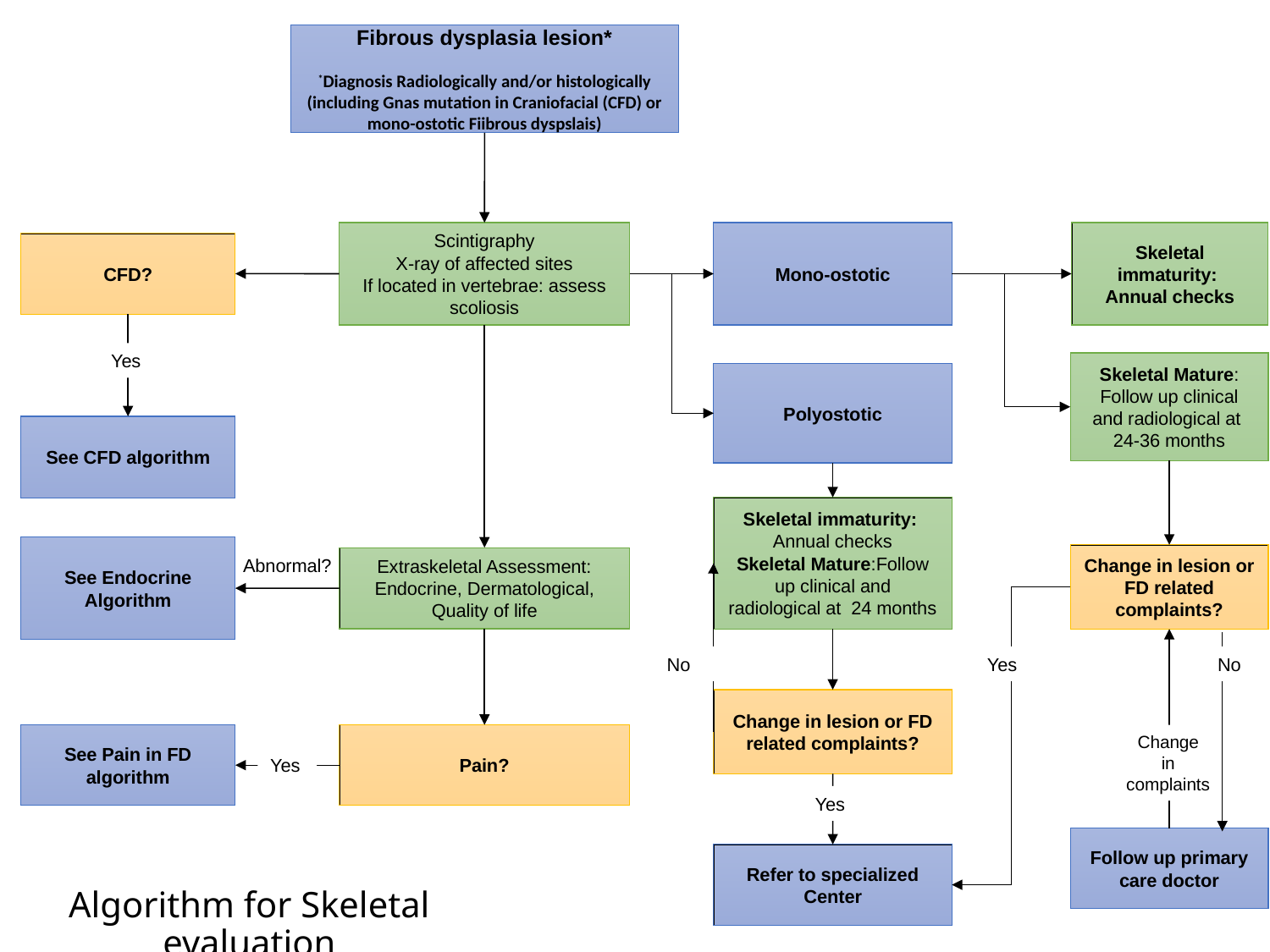

Fibrous dysplasia lesion*
*Diagnosis Radiologically and/or histologically (including Gnas mutation in Craniofacial (CFD) or mono-ostotic Fiibrous dyspslais)
Skeletal immaturity:
Annual checks
Scintigraphy
X-ray of affected sites
If located in vertebrae: assess scoliosis
Mono-ostotic
CFD?
Yes
Skeletal Mature:
Follow up clinical and radiological at 24-36 months
Polyostotic
See CFD algorithm
Skeletal immaturity:
Annual checks
Skeletal Mature:Follow up clinical and radiological at 24 months
See Endocrine
Algorithm
Change in lesion or FD related complaints?
Abnormal?
Extraskeletal Assessment:
Endocrine, Dermatological, Quality of life
No
Yes
No
Change in lesion or FD related complaints?
See Pain in FD algorithm
Pain?
Change
in
complaints
Yes
Yes
Follow up primary care doctor
Refer to specialized Center
Algorithm for Skeletal evaluation
