# Supplementary material for: Best practice management guidelines for fibrous dysplasia/McCune-Albright syndrome: a consensus statement from the FD/MAS international consortium
Source: Orphanet J Rare Dis. 2019 Jun 13;14:139. doi: 10.1186/s13023-019-1102-9 (PMC6567644; doi:10.1186/s13023-019-1102-9)
Supplement: Supplementary file 3 — Flowcharts Management of Bone Pain. (PPTX 45 kb) [file 13023_2019_1102_MOESM3_ESM.pptx]

## Slide 1
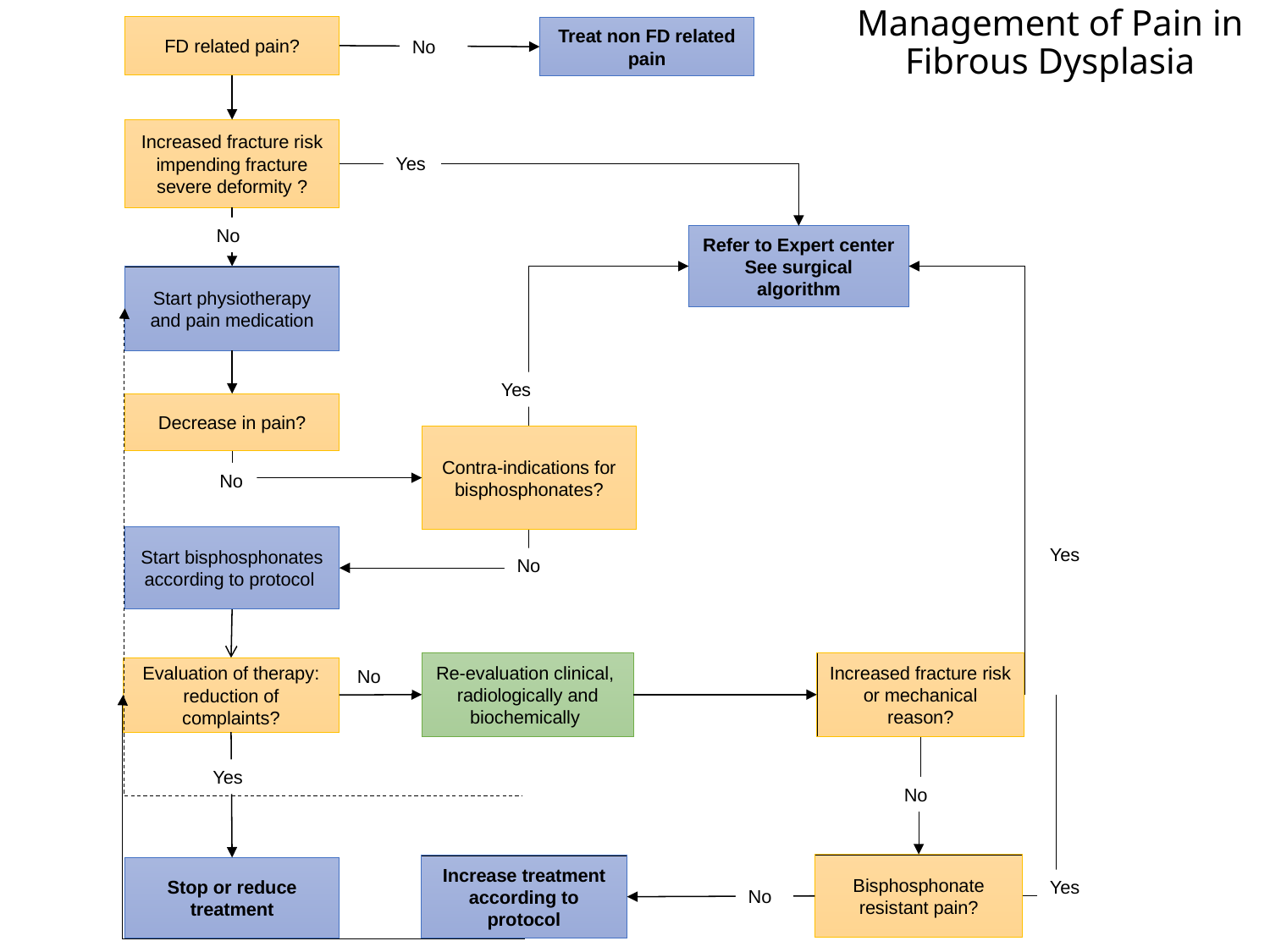

Management of Pain in
Fibrous Dysplasia
FD related pain?
Treat non FD related pain
No
Increased fracture risk impending fracture severe deformity ?
Yes
No
Refer to Expert center
See surgical algorithm
Start physiotherapy and pain medication
Yes
Decrease in pain?
Contra-indications for bisphosphonates?
No
Start bisphosphonates according to protocol
Yes
No
Re-evaluation clinical, radiologically and biochemically
Increased fracture risk or mechanical reason?
Evaluation of therapy: reduction of complaints?
No
Yes
No
Bisphosphonate resistant pain?
Increase treatment according to protocol
Stop or reduce treatment
Yes
No
