# Supplementary material for: Best practice management guidelines for fibrous dysplasia/McCune-Albright syndrome: a consensus statement from the FD/MAS international consortium
Source: Orphanet J Rare Dis. 2019 Jun 13;14:139. doi: 10.1186/s13023-019-1102-9 (PMC6567644; doi:10.1186/s13023-019-1102-9)
Supplement: Supplementary file 4 — Flowcharts MAS Endo. (PPTX 64 kb) [file 13023_2019_1102_MOESM4_ESM.pptx]

## Slide 1
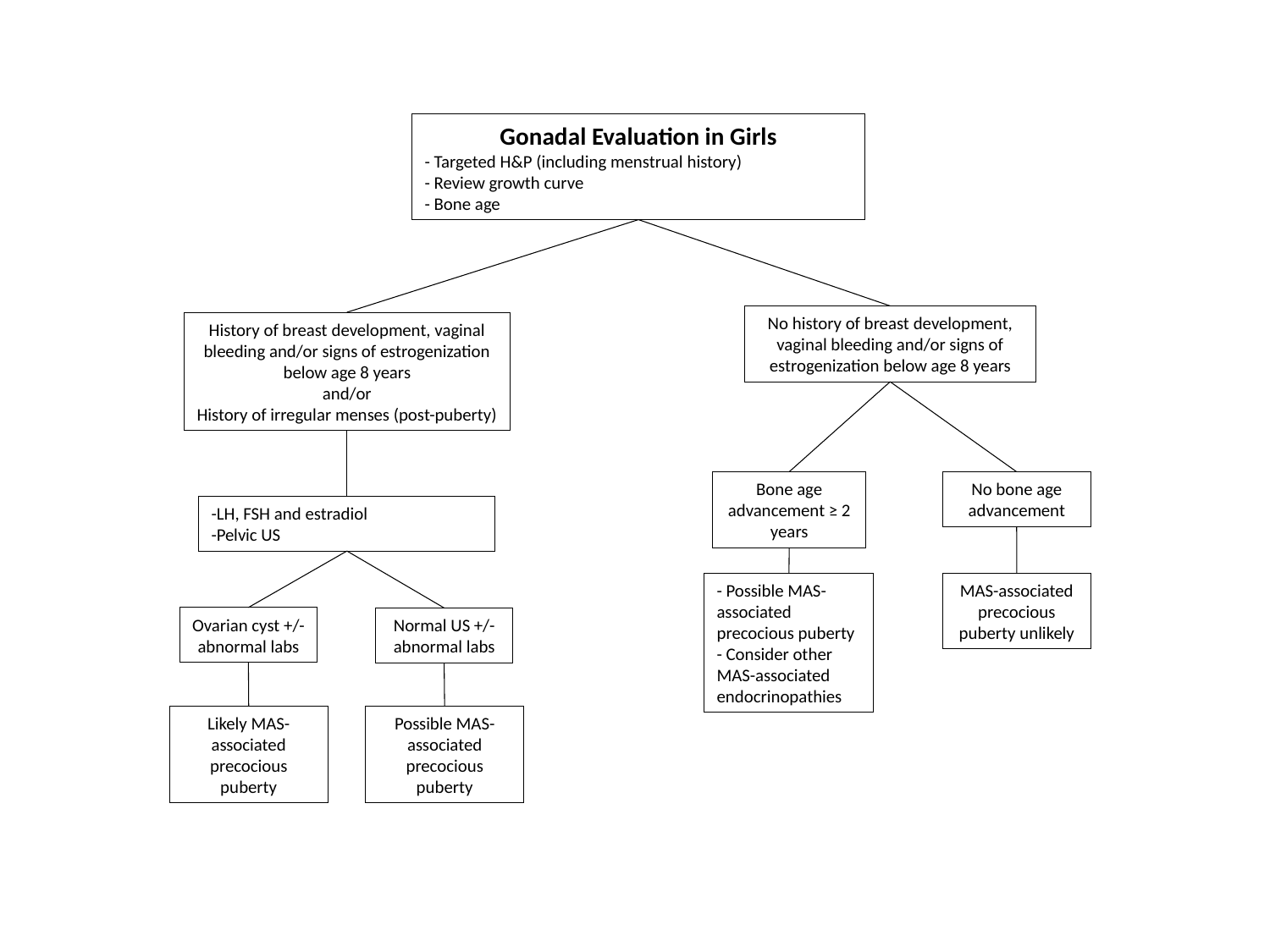

Gonadal Evaluation in Girls
- Targeted H&P (including menstrual history)
- Review growth curve
- Bone age
No history of breast development, vaginal bleeding and/or signs of estrogenization below age 8 years
History of breast development, vaginal bleeding and/or signs of estrogenization below age 8 years
and/or
History of irregular menses (post-puberty)
Bone age advancement ≥ 2 years
No bone age advancement
-LH, FSH and estradiol
-Pelvic US
- Possible MAS-associated precocious puberty
- Consider other MAS-associated endocrinopathies
MAS-associated precocious puberty unlikely
Ovarian cyst +/- abnormal labs
Normal US +/- abnormal labs
Likely MAS-associated precocious puberty
Possible MAS-associated precocious puberty

## Slide 2
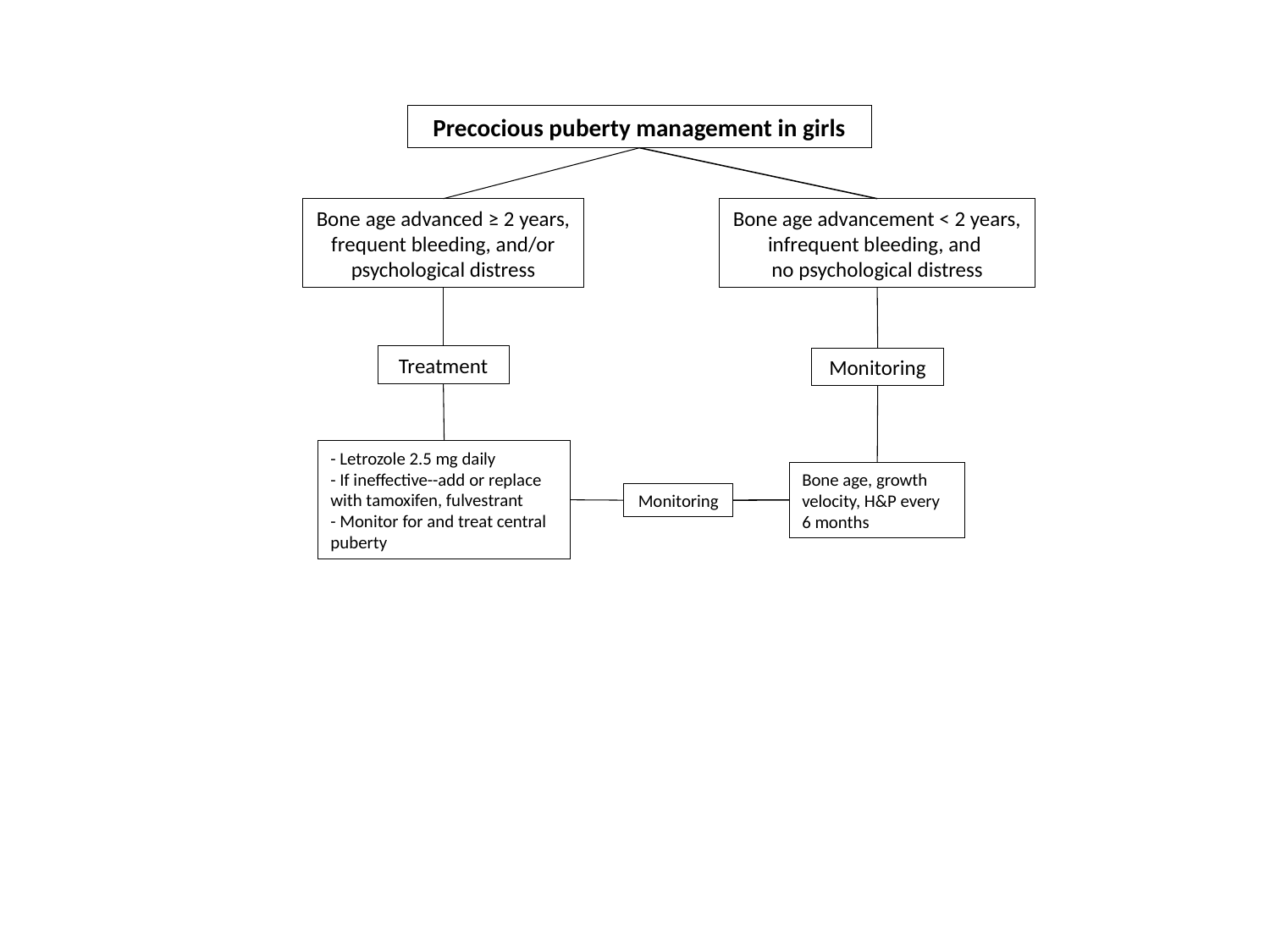

Precocious puberty management in girls
Bone age advancement < 2 years,
infrequent bleeding, and
no psychological distress
Bone age advanced ≥ 2 years, frequent bleeding, and/or psychological distress
Treatment
Monitoring
- Letrozole 2.5 mg daily
- If ineffective--add or replace with tamoxifen, fulvestrant
- Monitor for and treat central puberty
Bone age, growth velocity, H&P every 6 months
Monitoring

## Slide 3
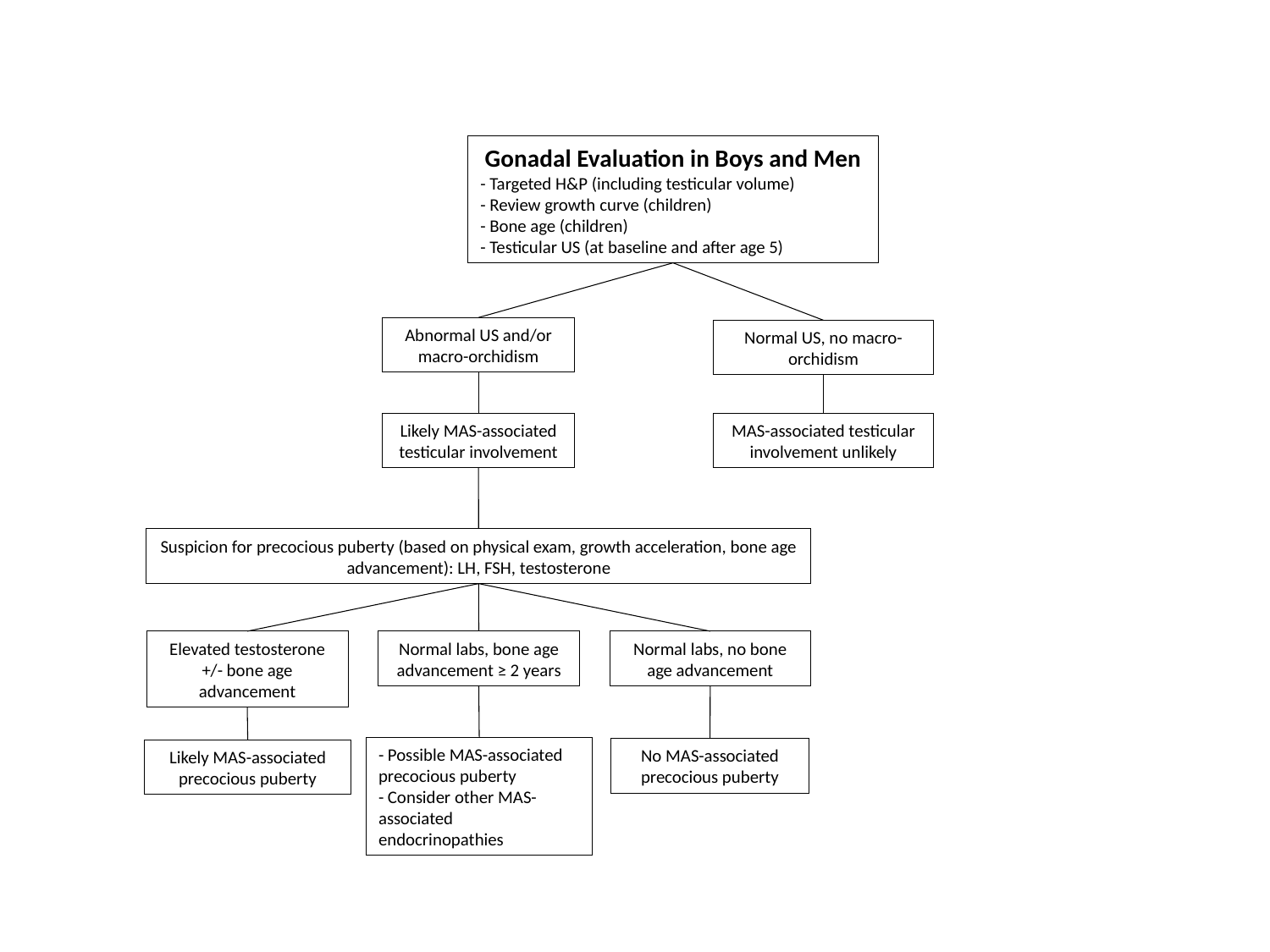

Gonadal Evaluation in Boys and Men
- Targeted H&P (including testicular volume)
- Review growth curve (children)
- Bone age (children)
- Testicular US (at baseline and after age 5)
Abnormal US and/or macro-orchidism
Normal US, no macro-orchidism
Likely MAS-associated testicular involvement
MAS-associated testicular involvement unlikely
Suspicion for precocious puberty (based on physical exam, growth acceleration, bone age advancement): LH, FSH, testosterone
Elevated testosterone +/- bone age advancement
Normal labs, bone age advancement ≥ 2 years
Normal labs, no bone age advancement
- Possible MAS-associated precocious puberty
- Consider other MAS-associated endocrinopathies
No MAS-associated precocious puberty
Likely MAS-associated precocious puberty

## Slide 4
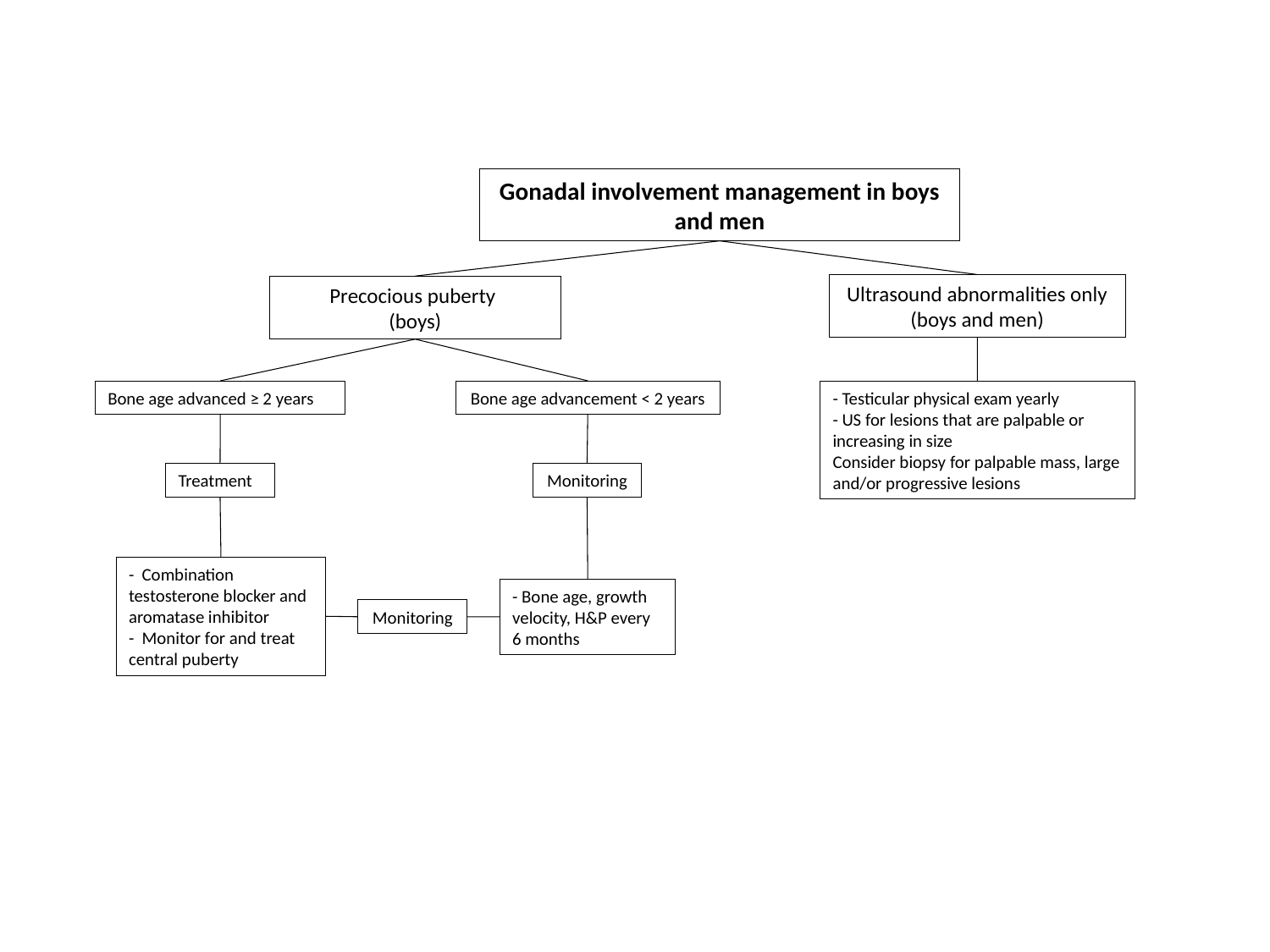

Gonadal involvement management in boys and men
Ultrasound abnormalities only (boys and men)
Precocious puberty
(boys)
Bone age advanced ≥ 2 years
- Testicular physical exam yearly
- US for lesions that are palpable or increasing in size
Consider biopsy for palpable mass, large and/or progressive lesions
Bone age advancement < 2 years
Treatment
Monitoring
- Combination testosterone blocker and aromatase inhibitor
- Monitor for and treat central puberty
- Bone age, growth velocity, H&P every 6 months
Monitoring

## Slide 5
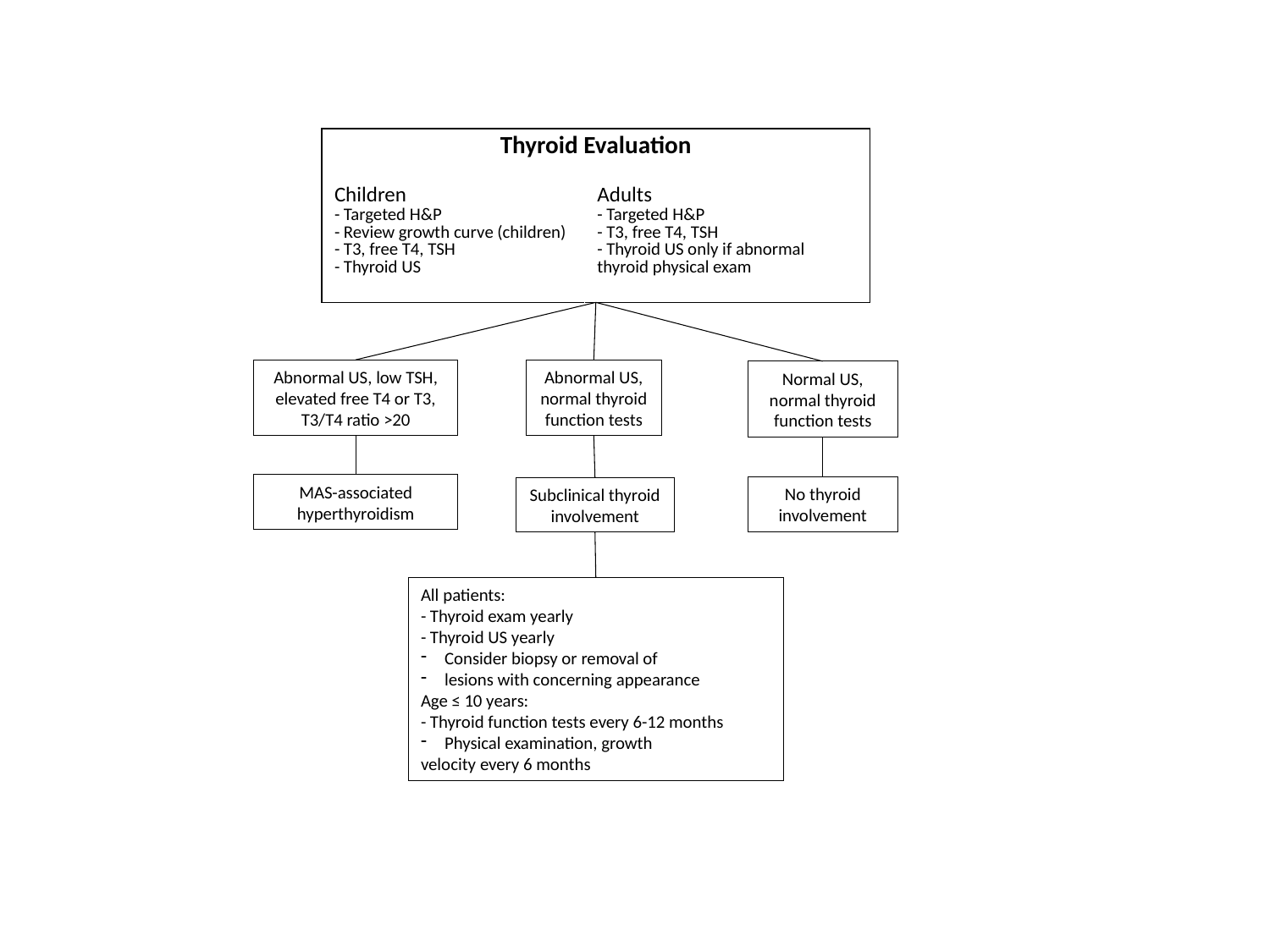

| Thyroid Evaluation | |
| --- | --- |
| Children - Targeted H&P - Review growth curve (children) - T3, free T4, TSH - Thyroid US | Adults - Targeted H&P - T3, free T4, TSH - Thyroid US only if abnormal thyroid physical exam |
Abnormal US, normal thyroid function tests
Abnormal US, low TSH, elevated free T4 or T3, T3/T4 ratio >20
Normal US, normal thyroid function tests
MAS-associated hyperthyroidism
No thyroid involvement
Subclinical thyroid involvement
All patients:
- Thyroid exam yearly
- Thyroid US yearly
Consider biopsy or removal of
lesions with concerning appearance
Age ≤ 10 years:
- Thyroid function tests every 6-12 months
Physical examination, growth
velocity every 6 months

## Slide 6
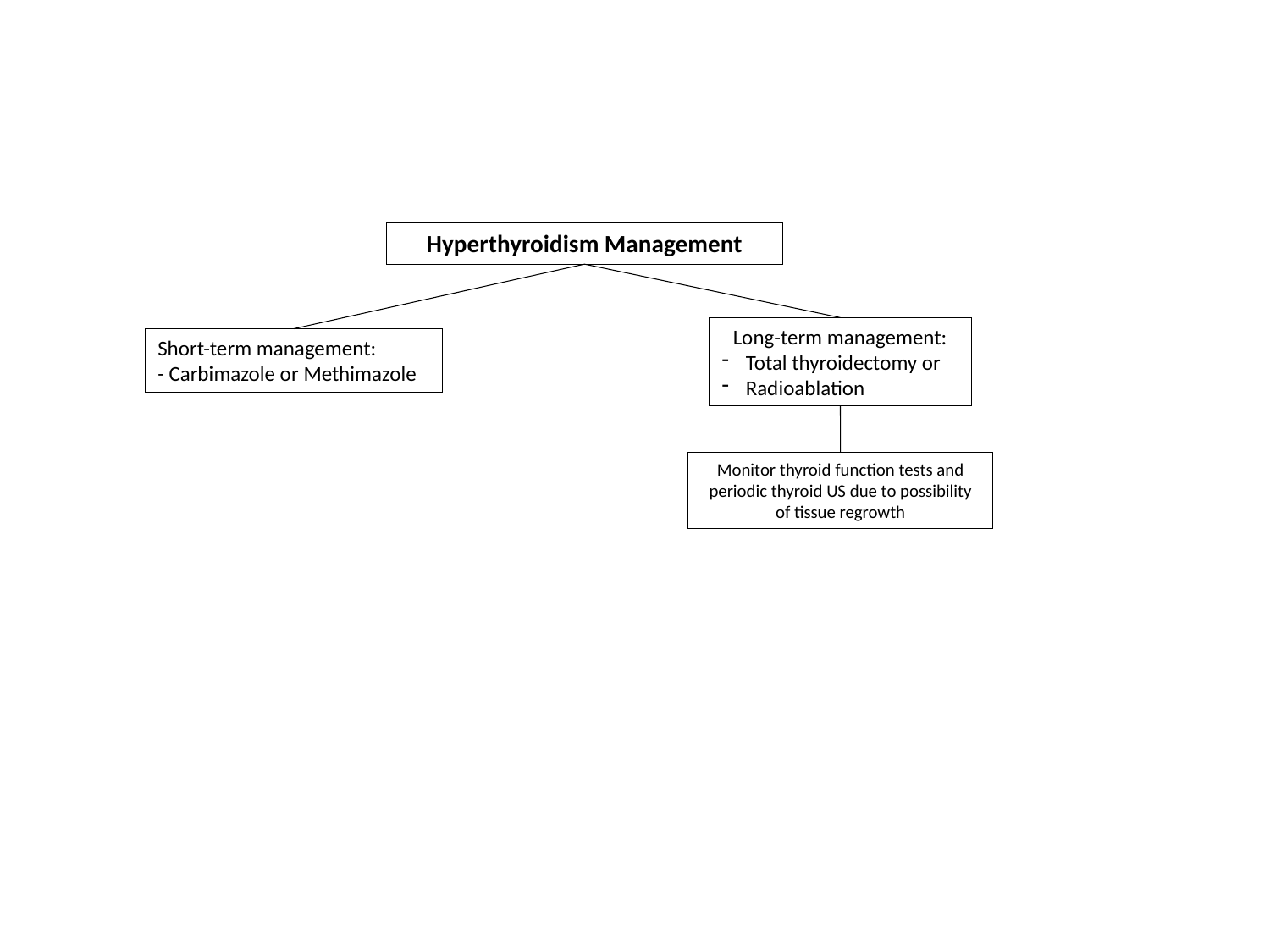

Hyperthyroidism Management
Long-term management:
Total thyroidectomy or
Radioablation
Short-term management:
- Carbimazole or Methimazole
Monitor thyroid function tests and periodic thyroid US due to possibility of tissue regrowth

## Slide 7
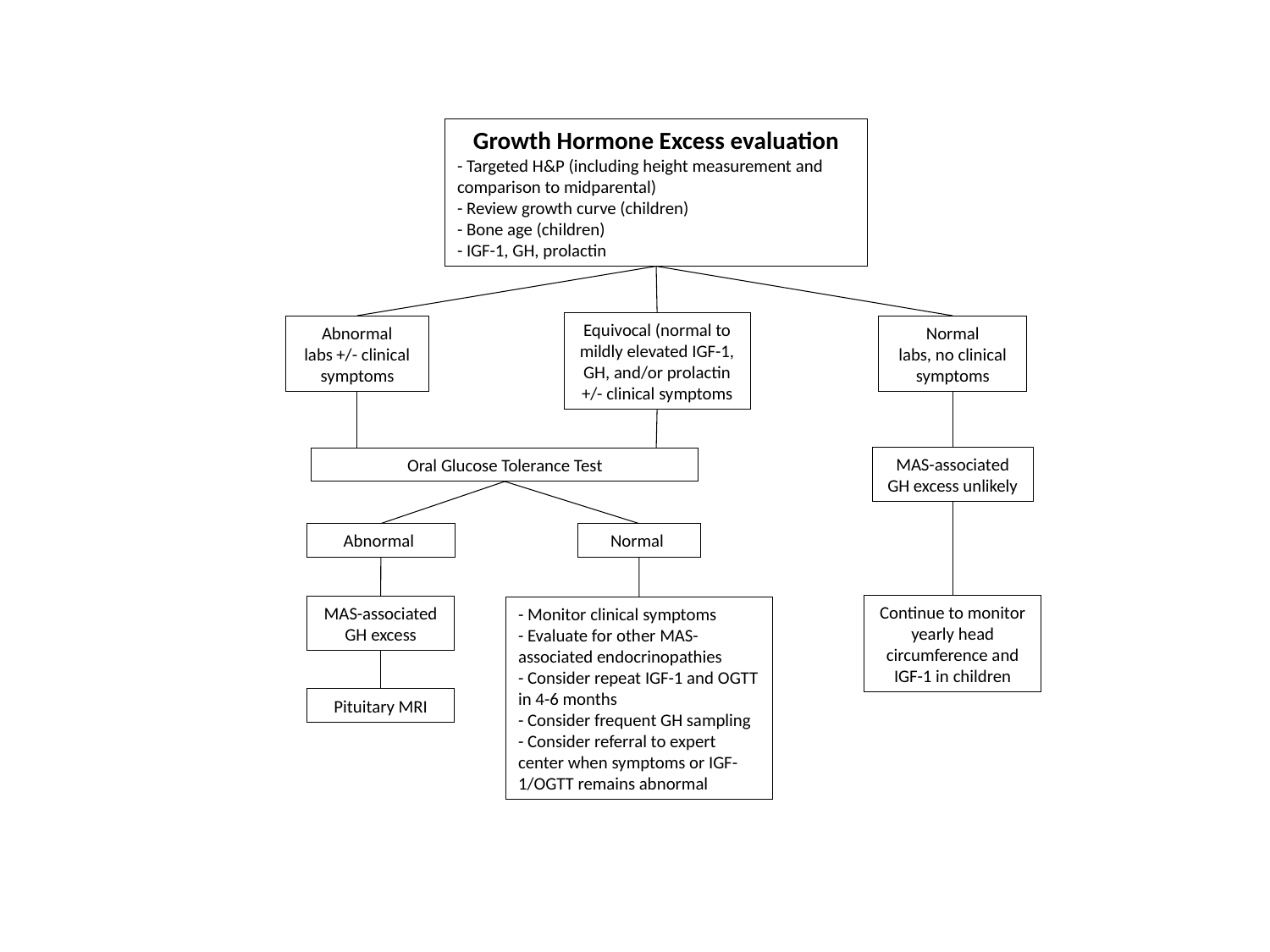

Growth Hormone Excess evaluation
- Targeted H&P (including height measurement and comparison to midparental)
- Review growth curve (children)
- Bone age (children)
- IGF-1, GH, prolactin
Equivocal (normal to mildly elevated IGF-1, GH, and/or prolactin +/- clinical symptoms
Abnormal
labs +/- clinical symptoms
Normal
labs, no clinical symptoms
MAS-associated GH excess unlikely
Oral Glucose Tolerance Test
Abnormal
Normal
Continue to monitor yearly head circumference and IGF-1 in children
MAS-associated GH excess
- Monitor clinical symptoms
- Evaluate for other MAS-associated endocrinopathies
- Consider repeat IGF-1 and OGTT in 4-6 months
- Consider frequent GH sampling
- Consider referral to expert center when symptoms or IGF-1/OGTT remains abnormal
Pituitary MRI

## Slide 8
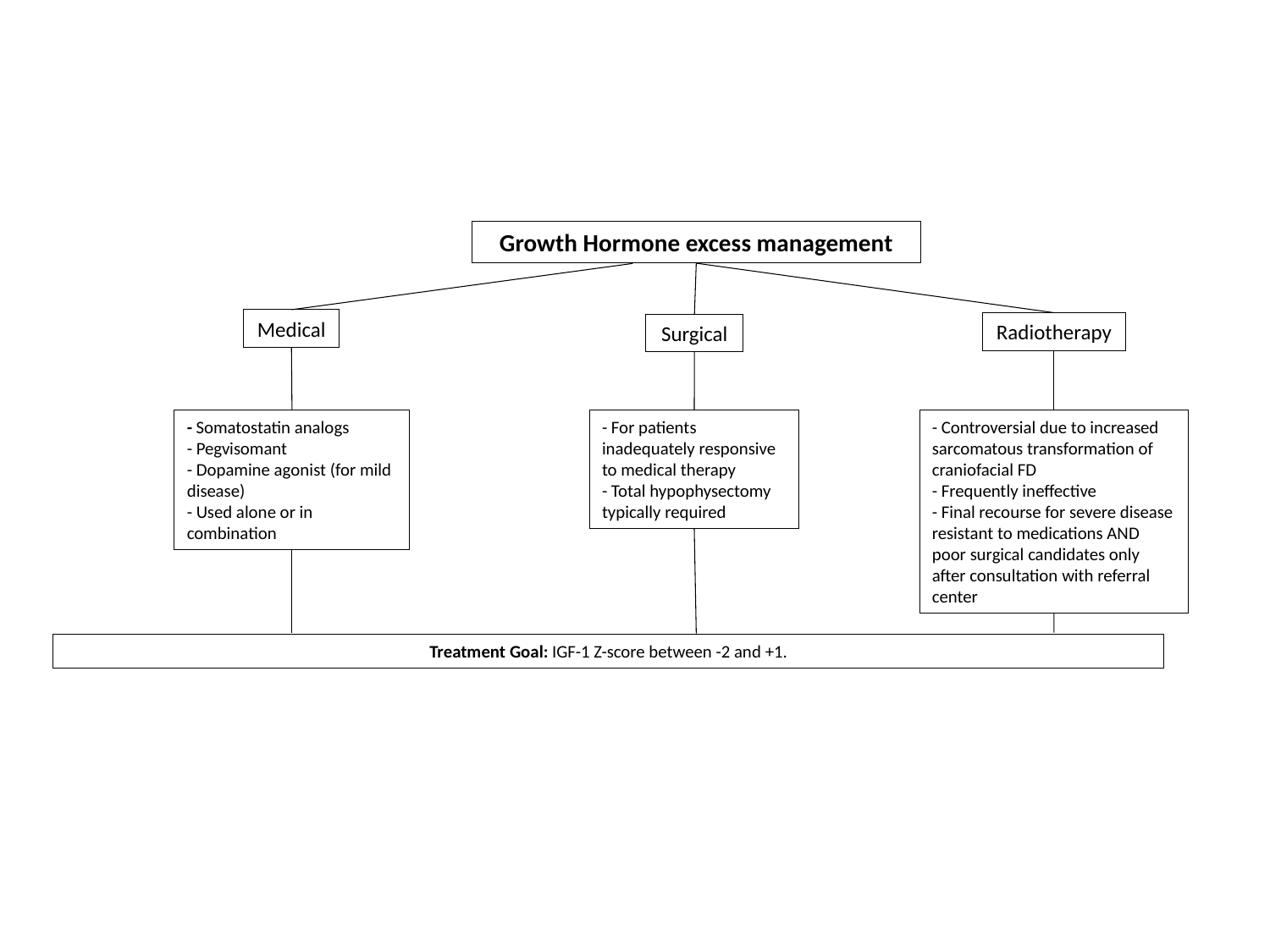

Growth Hormone excess management
Medical
Radiotherapy
Surgical
- Somatostatin analogs
- Pegvisomant
- Dopamine agonist (for mild disease)
- Used alone or in combination
- For patients inadequately responsive to medical therapy
- Total hypophysectomy typically required
- Controversial due to increased sarcomatous transformation of craniofacial FD
- Frequently ineffective
- Final recourse for severe disease resistant to medications AND poor surgical candidates only after consultation with referral center
Treatment Goal: IGF-1 Z-score between -2 and +1.

## Slide 9
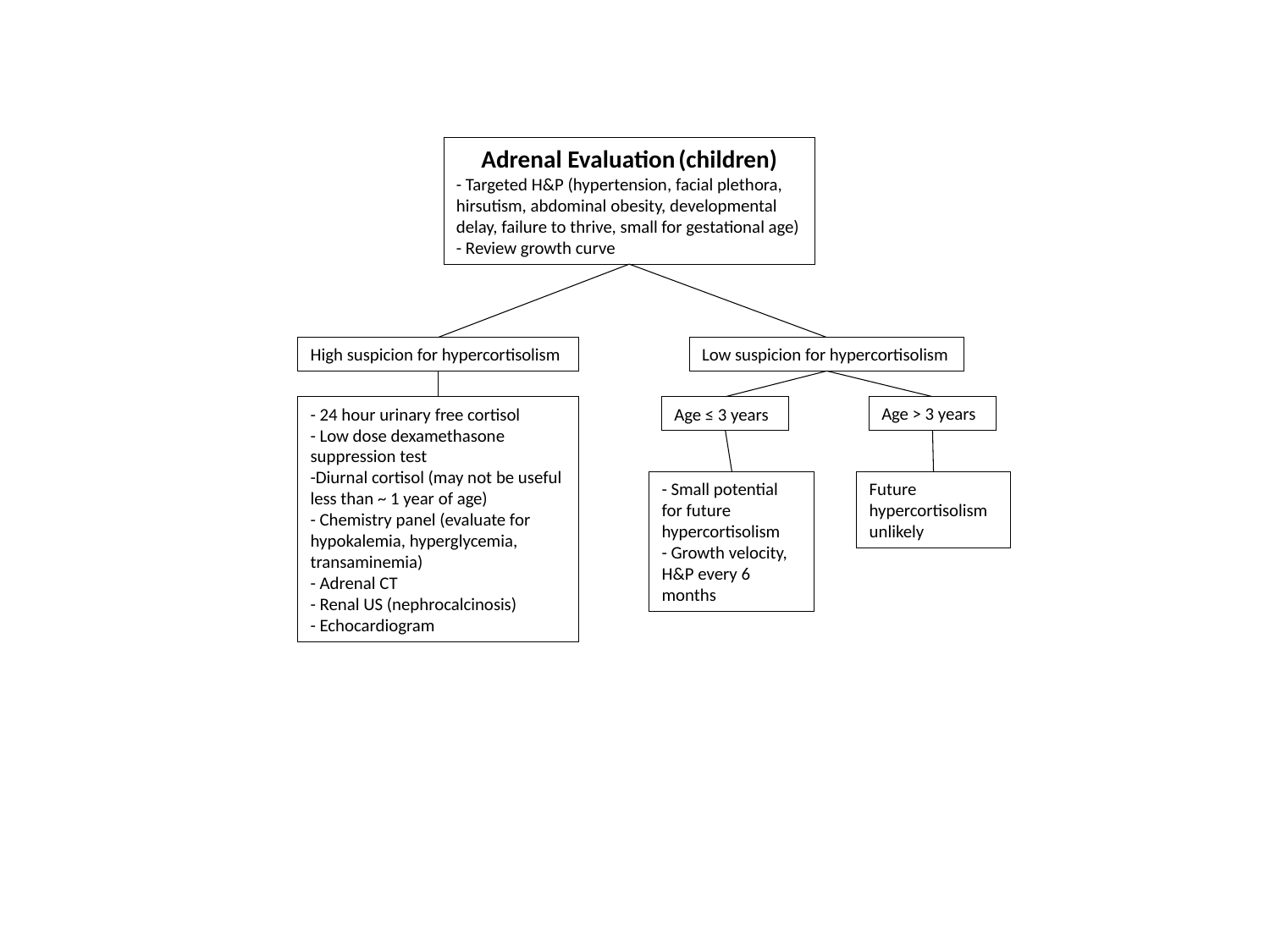

Adrenal Evaluation (children)
- Targeted H&P (hypertension, facial plethora, hirsutism, abdominal obesity, developmental delay, failure to thrive, small for gestational age)
- Review growth curve
Low suspicion for hypercortisolism
High suspicion for hypercortisolism
Age > 3 years
- 24 hour urinary free cortisol
- Low dose dexamethasone suppression test
-Diurnal cortisol (may not be useful less than ~ 1 year of age)
- Chemistry panel (evaluate for hypokalemia, hyperglycemia, transaminemia)
- Adrenal CT
- Renal US (nephrocalcinosis)
- Echocardiogram
Age ≤ 3 years
- Small potential for future hypercortisolism
- Growth velocity, H&P every 6 months
Future hypercortisolism unlikely

## Slide 10
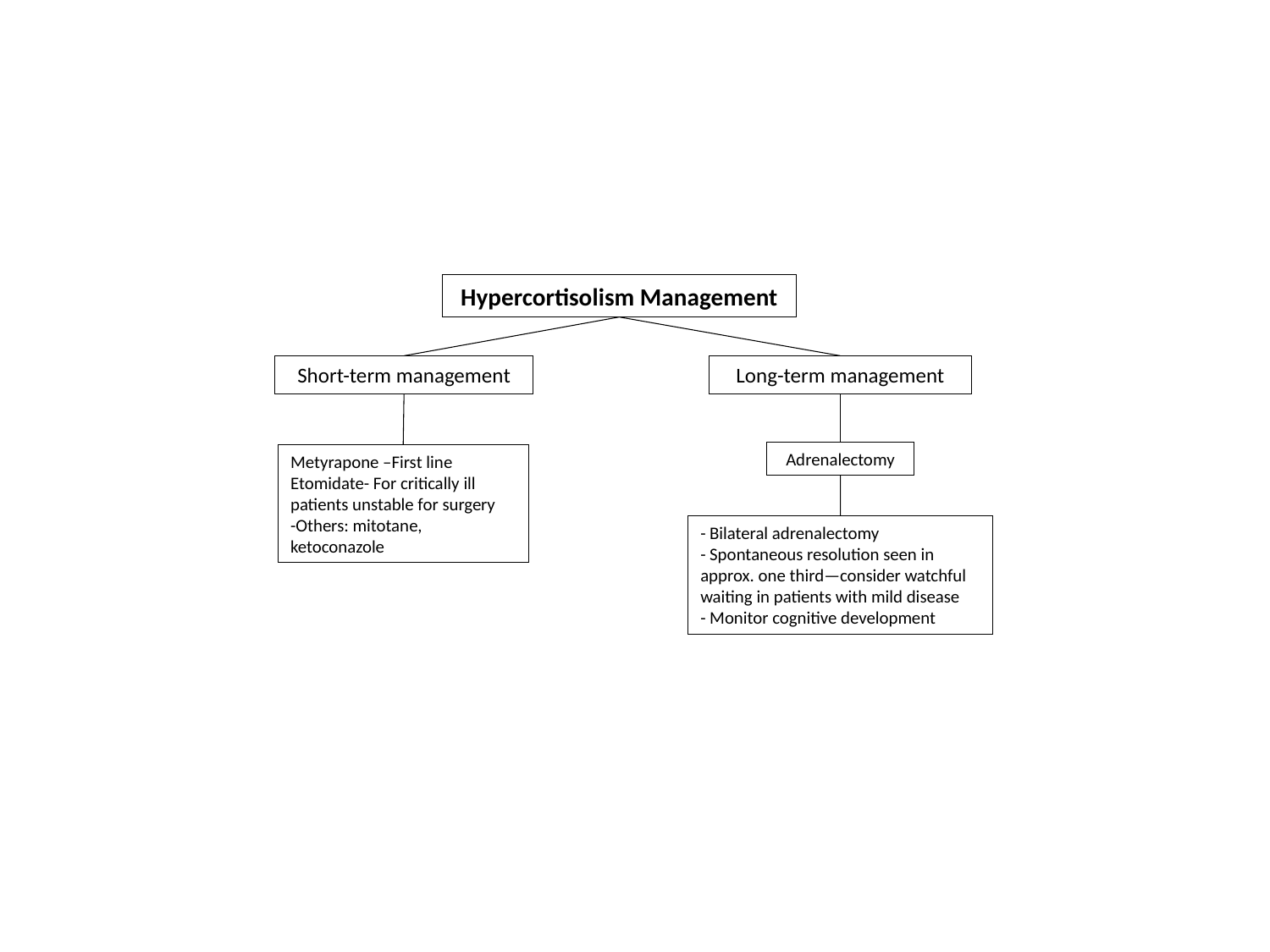

Hypercortisolism Management
Short-term management
Long-term management
Adrenalectomy
Metyrapone –First line
Etomidate- For critically ill patients unstable for surgery
-Others: mitotane, ketoconazole
- Bilateral adrenalectomy
- Spontaneous resolution seen in approx. one third—consider watchful waiting in patients with mild disease
- Monitor cognitive development
