# Supplementary figures and images for: Best practice management guidelines for fibrous dysplasia/McCune-Albright syndrome: a consensus statement from the FD/MAS international consortium
Source: Orphanet J Rare Dis. 2019 Jun 13;14:139. doi: 10.1186/s13023-019-1102-9 (PMC6567644; doi:10.1186/s13023-019-1102-9)

## Algorithm surgical approach femur

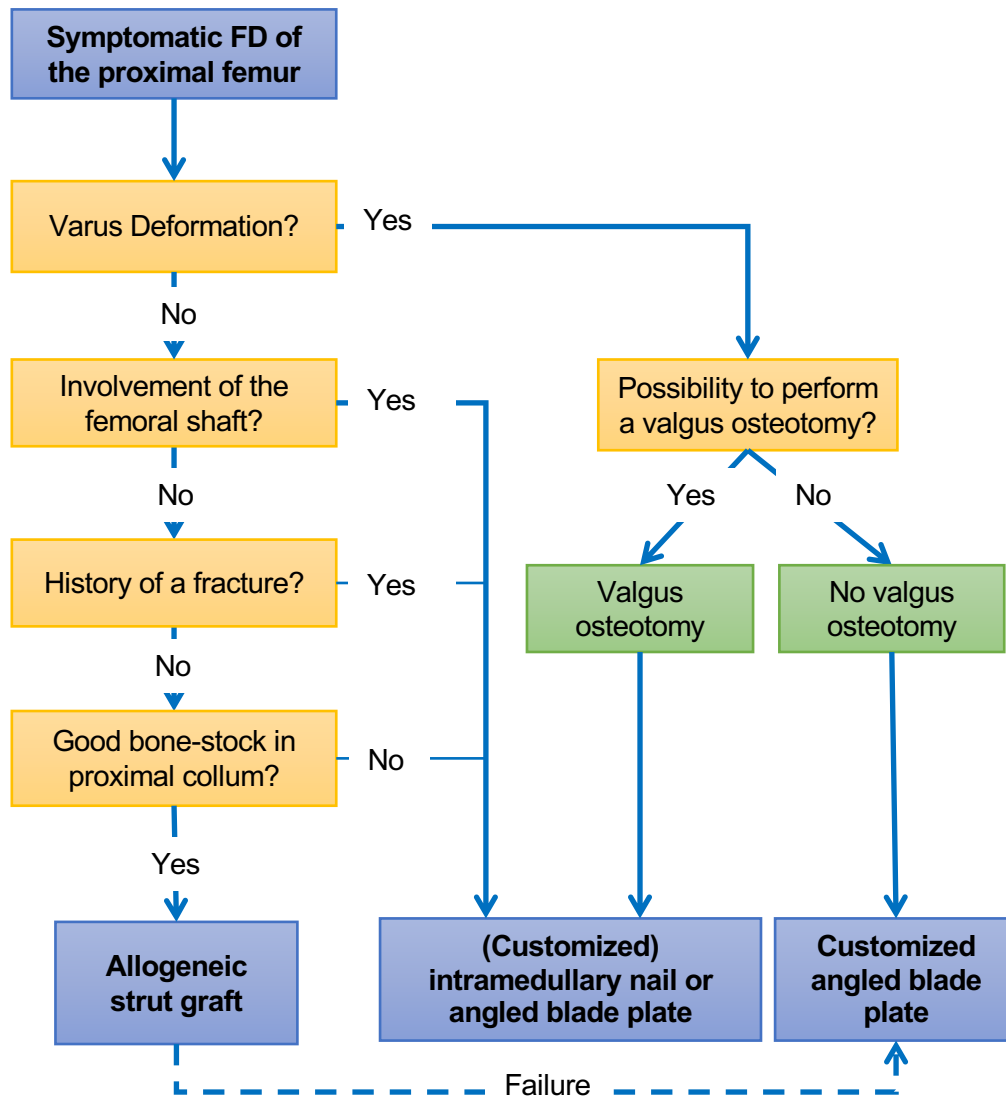

Supplement: Supplementary file 5 — Flowcharts Surgical Management of Proximal Femur. (PDF 34 kb) [file 13023_2019_1102_MOESM5_ESM.pdf]
