# Supplementary material for: Best practice management guidelines for fibrous dysplasia/McCune-Albright syndrome: a consensus statement from the FD/MAS international consortium
Source: Orphanet J Rare Dis. 2019 Jun 13;14:139. doi: 10.1186/s13023-019-1102-9 (PMC6567644; doi:10.1186/s13023-019-1102-9)
Supplement: Supplementary file 6 — Flowcharts Management of CFFD. (PPTX 36 kb) [file 13023_2019_1102_MOESM6_ESM.pptx]

## Slide 1
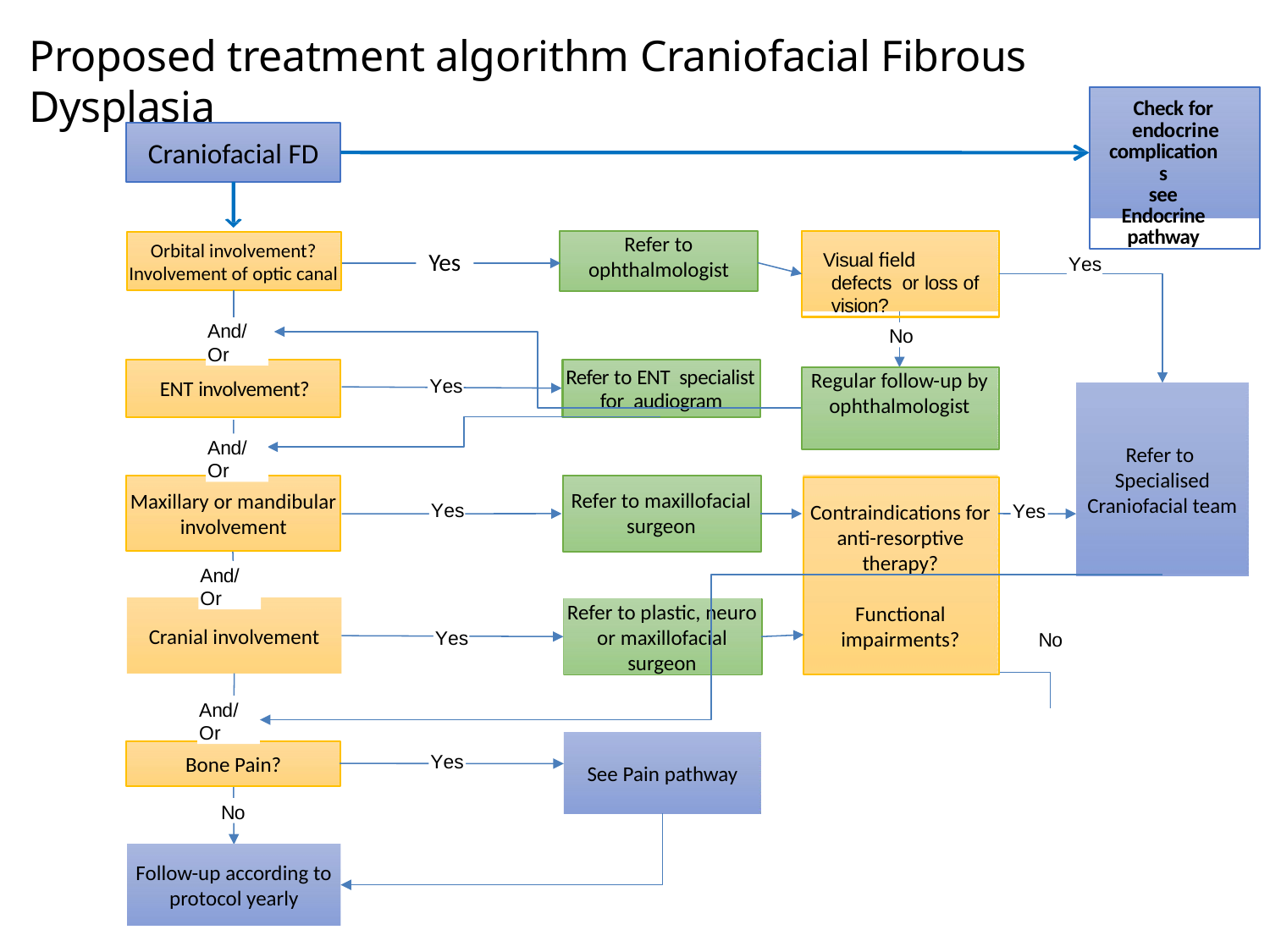

# Proposed treatment algorithm Craniofacial Fibrous Dysplasia
Check for endocrine
complications
see Endocrine
pathway
Craniofacial FD
Visual field defects or loss of vision?
Refer to ophthalmologist
Orbital involvement? Involvement of optic canal
Yes
Yes
And/Or
No
Refer to ENT specialist for audiogram
Regular follow-up by ophthalmologist
Yes
ENT involvement?
Refer to
Specialised Craniofacial team
And/Or
Refer to maxillofacial surgeon
Maxillary or mandibular involvement
Contraindications for anti-resorptive therapy?
Functional impairments?
Yes
Yes
And/Or
Cranial involvement
Refer to plastic, neuro or maxillofacial surgeon
Yes
No
And/Or
See Pain pathway
Bone Pain?
Yes
No
Follow-up according to protocol yearly
